# Reduced lung metastasis in endothelial cell-specific transforming growth factor β type II receptor-deficient mice with decreased CD44 expression

**DOI:** 10.1016/j.isci.2024.111502

**Published:** 2024-11-28

**Authors:** Kako Hanada, Yuki Saito, Takahiro Takagi, Mitsuki Go, Yota Nakano, Toshihiko Inagawa, Hideyo Hirai, Marcus Fruttiger, Susumu Itoh, Fumiko Itoh

**Affiliations:** 1 Laboratory of Stem Cell Regulation, Tokyo University of Pharmacy and Life Sciences, 1432-1 Horinouchi, Hachioji, Tokyo 192-0392, Japan; 2 Laboratory of Cardiovascular Medicine, Tokyo University of Pharmacy and Life Sciences, 1432-1 Horinouchi, Hachioji, Tokyo 192-0392, Japan; 3 Laboratory of Biochemistry, Showa Pharmaceutical University, 3-3165 Higashi-Tamagawagakuen, Machida, Tokyo 194-8543, Japan; 4 UCL Institute of Ophthalmology, University College London, 11-43 Bath Street, London EC1V 9EL, UK

**Keywords:** Natural sciences, Biological sciences, Systems biology, Cancer systems biology, Cancer

## Abstract

Transforming growth factor β (TGF-β) is abundantly present in the tumor microenvironment, contributing to cancer progression. However, the regulatory mechanism by which TGF-β affects vascular endothelial cells (ECs) in the tumor microenvironment is not well understood. Herein, we generated tamoxifen-inducible TGF-β type II receptor (*TβRII*) knockout mice, specifically targeting ECs (TβRII^iΔEC^), by crossbreeding TβRII-floxed mice with Pdgfb-icreER mice. We established tumor-bearing mice by transplanting Lewis lung carcinoma (LLC) cells. TβRII^iΔEC^ mice exhibited increased tumor angiogenesis with fragile new blood vessels, increased bleeding, and hypoxia compared to control mice. Consequently, the compromised tumor microenvironment precipitated a notable surge in circulating tumor cells. Paradoxically, lung metastasis showed a significant decline. This intriguing discrepancy was explained by a reduction in the engraftment between cancer cells and ECs. Disruption of TGF-β signaling downregulated CD44 on ECs, hindering cancer cell adhesion. These findings highlight TGF-β′s role in promoting metastasis by modulating EC function.

## Highlights

• Endothelial-specific TGF-β receptor knockout leads to fragile tumor vasculature
• Loss of endothelial TGF-β signaling reduces lung metastasis in tumor-bearing mice
• TβRII deletion in tumor endothelial cells reduces CD44 expression and increases CTCs
• CD44 downregulation inhibits cancer cell adhesion to endothelial cells

## Graphical abstract

**Figure:**
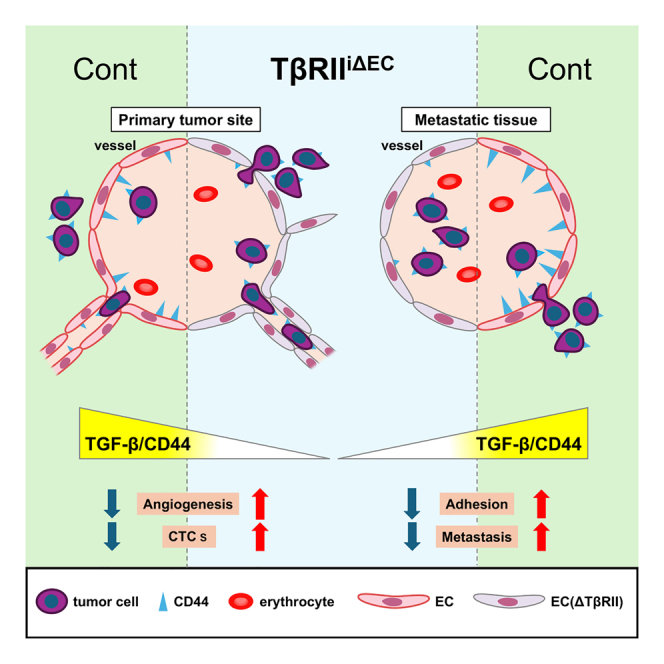

## Introduction

Rapidly proliferating tumors necessitate an augmented vascular network to meet heightened demands for nutrients and oxygen.^1^^,^^2^ Consequently, cancer cells, in tandem with the hypoxic microenvironment enveloping them, generate angiogenic factors that instigate the proliferation of blood vessels.^3^ However, in stark contrast to vessels in healthy tissues, tumor-associated vasculature exhibits marked dysfunction, characterized by a disorganized network of permeable, convoluted, and heterogeneous endothelial tubules lacking pericyte support. These vascular anomalies contribute to compromised blood flow, heightened vascular permeability, and inadequate perfusion within the tumor microenvironment.^4^ Recognizing the importance of normalizing angiogenesis, particularly in the context of optimizing drug delivery to tumors, has become a focal point in contemporary cancer therapeutics.^5^^,^^6^

Transforming growth factor β (TGF-β) signaling plays a biphasic role in cancer progression, acting as both a tumor suppressor and promoter depending on the stage of tumorigenesis.^7^ TGF-βs elicit their cellular effects by binding to specific type I and type II serine/threonine kinase receptors known as the TGF-β type I (TβRI; also termed activin receptor-like kinase-5 [ALK5]) and type II receptors (TβRII), respectively. Upon ligand binding, constitutively active TβRII phosphorylates ALK5. After phosphorylation of ALK5 by TβRII, ALK5 kinase becomes active. Subsequently, ALK5 kinase phosphorylates two serine residues in receptor-regulated Smads (R-Smads; i.e., Smad2 and Smad3) at the extreme carboxyl terminal. Then, the two activated R-Smads form a complex with a common partner Smad, Smad4, and they accumulate in the nucleus where they act as transcriptional regulators to govern TGF-β target genes.^8^^,^^9^^,^^10^^,^^11^ In early-stage cancer, TGF-β suppresses tumor growth by inducing cell-cycle arrest and apoptosis via the ALK5-Smad2/3 pathway. In contrast, in advanced stages, tumor cells evade these inhibitory effects and exploit TGF-β to promote epithelial-to-mesenchymal transition (EMT), invasion, and metastasis.^12^^,^^13^ Additionally, TGF-β contributes to the creation of an immunosuppressive tumor microenvironment by recruiting regulatory T cells and myeloid-derived suppressor cells.^14^^,^^15^^,^^16^

In endothelial cells (ECs), TGF-β signaling operates through two distinct pathways with opposing effects on angiogenesis. The ALK5-SMAD2/3 pathway maintains vascular stability and inhibits excessive endothelial proliferation, while the ALK1-SMAD1/5 pathway promotes endothelial proliferation and migration.^17^ In the tumor microenvironment, this balance is disrupted, with the overactivation of the ALK1-Smad1/5 pathway, leading to aberrant angiogenesis characterized by abnormal, leaky vessels that contribute to hypoxia, tumor growth, and metastasis.^18^

The critical role of TGF-β signaling in endothelial cells and angiogenesis has been demonstrated in several genetically modified mouse models.^17^ Notably, TβRII- or ALK5-deficient mice displayed embryonic lethality attributed to angiogenic defects on day E10.5. Additionally, the inhibition of TGF-β signaling through endothelial-specific Smad2 deficiency and systemic Smad3 deficiency showed embryonic lethality despite the maintenance of a vascular network.^19^ These findings underscore the crucial role of TGF-β signals in endothelial cells (ECs) for vascular development; however, its precise role in tumor angiogenesis, particularly in adulthood, remains to be fully elucidated.

Angiogenesis, the formation of new blood vessels from pre-existing ones, is essential not only during embryogenesis but also throughout adulthood.^20^ During angiogenesis, ECs migrate and proliferate in response to various angiogenic agonists, such as vascular endothelial growth factor (VEGF), fibroblast growth factor 2 (FGF-2), and angiopoietin. In tumors, this process is dysregulated, resulting in abnormal vasculature characterized by disorganization, increased permeability, and inefficient blood flow. This creates a hypoxic environment that promotes tumor progression and metastasis.^20^^,^^21^

A critical early event in cancer metastasis involves the interaction between cancer cells and ECs, where CD44 and its ligand hyaluronic acid (HA) play key roles. CD44, a cell surface glycoprotein, mediates adhesion and communication between cancer cells and vascular ECs.^22^^,^^23^ The binding of CD44 to HA promotes the adherence of cancer cells to the endothelial lining, initiating a series of events that culminate in extravasation—the process through which cancer cells exit the bloodstream and infiltrate surrounding tissues. This CD44-HA interaction is a central role in hematogenous tumor metastasis, shaping the metastatic potential of cancer cells.^24^

In this study, we employed genetic approaches with mice in which the *TβRII* gene is conditionally deleted in ECs using Pdgfb-icreER transgenic mice.^25^ We demonstrated that endothelial TβRII deletion in adult mice does not affect tumor growth but increases tumor angiogenesis. Inhibition of TGF-β signaling in tumor vessels resulted in highly permeable vessels, exacerbating the hypoxic condition of the cancer microenvironment and augmenting tumor circulating cells (CTCs). These results suggest that endogenous TGF-β signaling promotes tumor vascular maturation and reduces tumor metastasis in the primary tumor, but enhances cancer cell receptivity in vascular ECs in metastatic organs. Ultimately, this suggests that endogenous TGF-β signaling may promote cancer metastasis.

## Results

### Targeted deletion of the transforming growth factor β type II receptor gene in endothelial cells has no impact on tumor growth

In the advanced stages of tumorigenesis, TGF-β promotes malignancy through various mechanisms, including the induction of the epithelial-mesenchymal transition (EMT), enhancement of the invasive and metastatic potential, facilitation of immune evasion, and involvement in the remodeling of the extracellular matrix.^16^ Whilst cancer cells autonomously produce TGF-β, actively contributing to the progression of malignancy,^26^ the precise impact of elevated TGF-β levels on tumor angiogenesis remains elusive. To elucidate the role of TGF-β in ECs, we generated mice with the tamoxifen-inducible deletion of the TGF-β type II receptor (*TβRII*) gene in ECs: TβRII-floxed (TβRII^fl/fl^) mice were crossed with platelet-derived growth factor β (Pdgfb)-iCreER mice^25^ to create TβRII^fl/fl^; Pdgfb-icreER (TβRII^iΔEC^) mice. Administration of tamoxifen caused an endothelial cell-specific loss of TβRII (Figures S1A, S1B, and S2) and a concomitant decrease in phosphorylated Smad2 (Figures S1C and S1D). Notably, the deletion of TGF-β/TβRII signaling had no discernible impact on the vasculature of adult mice, as evidenced by the viability and health of TβRII^iΔEC^ mice after 6 months of tamoxifen treatment (data not shown).

**Figure 1:**
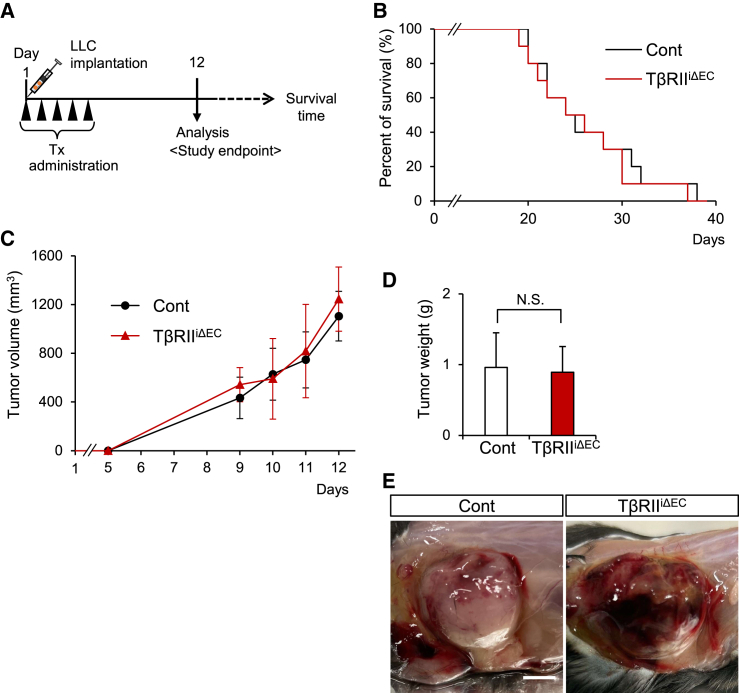
Impact of selective inhibition of TβRII signaling on tumor growth (A) Experimental protocol for tamoxifen (Tx) injection and LLC implantation. (B) Survival curve depicts mice in the control (Cont) and TβRII^iΔEC^ tumor-bearing groups (each *n* = 10). (C) Tumor volume curve of these respective groups (*n* = 10 mice/group). Data are presented as means ± SDs. (D) Excised tumor weights of these mice at the endpoint (day 12). Data are presented as means ± SDs. (unpaired two-tailed t-test, *n* = 6 mice/group, N.S.: not significant). (E) Representative photographic images of tumors of the control and TβRII^iΔEC^ mice at day 12. Scale bars: 5 mm.

To assess the impact of TGF-β within the tumor vasculature, we subcutaneously transplanted Lewis lung carcinoma (LLC) cells into control (Cont) or TβRII^iΔEC^-recipient mice upon the initiation of tamoxifen administration (Figure 1A). The persistence times of the tumor-bearing mice showed no significant difference between the control and the TβRII^iΔEC^ mice (Figure 1B), and neither did the tumor size over the course of 12 days postimplantation, nor the tumor weight at the study endpoint (Figures 1C and 1D). However, the gross appearance of the tumors in the TβRII^iΔEC^ mice exhibited notably higher vascularity when compared to that in control mice at the study endpoint (Figure 1E). These findings suggest that tumors in TβRII^iΔEC^ mice may develop leaky vessels and/or undergo increased angiogenesis.

### Transforming growth factor β type II receptor^iΔEC^ mice had increased tumor angiogenesis

**Figure 2:**
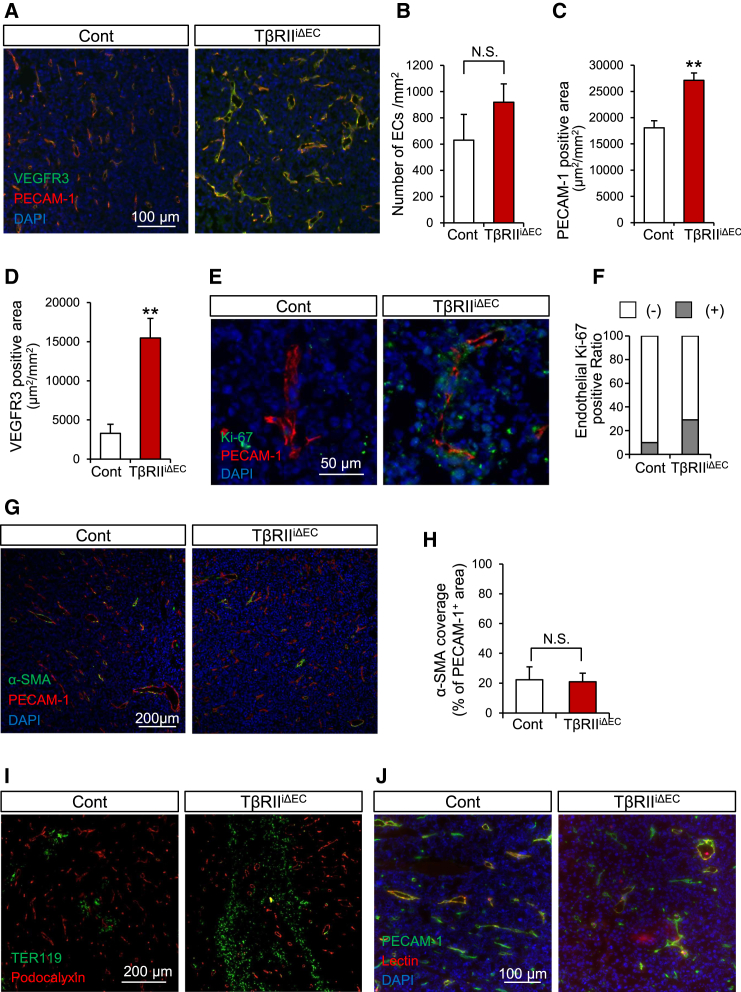
Impact of endothelial TβRII deficiency on tumor angiogenesis (A) Immunofluorescence analysis of tumor sections at day 12. (B–D) Quantitative analysis of images from 3 mice, including the representative image shown in (A). The number of blood vessels (B), PECAM-1-positive area (C), and VEGFR3-positive area (D) were analyzed. Data are presented as means ± SDs. Statistical comparisons were performed using an unpaired two-tailed t-test. *n* = 3 mice/group, with 2 images (1 mm^2^ each) analyzed per mouse. N.S.: not significant (B); ∗∗*p* < 0.01 (C and D). Measurements were performed using KEYENCE BZ-X800 Analyzer. (E and F) Immunofluorescence analysis of tumor sections (E). The proportion of Ki-67-positive cells among PECAM-1-positive cells was counted on the basis of the image in E. The proportion of Ki-67-positive cells among PECAM-1-positive cells was counted on the basis of the image in E (F). (G and H) Immunofluorescence analysis of tumor sections (G). The percentage of α-SMA cells among PECAM1-positive cells was counted on the basis of the image in (G). Data are presented as means ± SDs. Statistical comparisons were performed using an unpaired two-tailed t-test. *n* = 3 mice/group, with 2 images (1 mm^2^ each) analyzed per mouse. N.S.: not significant. (H). (I and J) Vascular leakiness was detected by immunofluorescence analysis. Leakage of red blood cells (TER119; green) and tomato-lectin (red) was confirmed to be greater in the TβRII^iΔEC^ mice than in the control mice.

We subsequently conducted immunofluorescence analysis to meticulously examine tumors in both the control and the TβRII^iΔEC^ mice at the study endpoints. PECAM-1 staining of tumor sections revealed that the quantity of tumor vessels per unit area remained consistent in the TβRII^iΔEC^ mice when compared with that in the control mice (Figures 2A and 2B). Nonetheless, there was a notable increase in the area of PECAM1-positive ECs and VEGFR3-positive lymph vessels, indicative of heightened angiogenesis (Figures 2C and 2D). To determine whether the increased vascularization stemmed from the alleviation of cell proliferation inhibited by TGF-β signals, the sections were costained with anti-PECAM-1 and anti-Ki-67, proliferation marker, antibodies. The merging frequency between the nucleus and Ki-67 in PECAM-1-positive cells was significantly elevated in the TβRII^iΔEC^ mice (Figures 2E and 2F). The healthy blood vessels are tightly covered with pericytes in general, whereas the newly formed vessels within the tumors in both the control and the TβRIIiΔEC mice extremely exhibited a reduction in α-smooth muscle actin (α-SMA)-positive pericytes coverage (Figures 2G and 2H). However, the degree of coverage did not differ between the two groups. Although pericyte coverage was unchanged between the control and the TβRII^iΔEC^ mice in both tumor-bearing cohorts, abundant red blood cells were found within the cancer tissues of TβRII^iΔEC^ mice, having leaked from podocalyxin-positive tumor blood vessels, indicating vessel fragility (Figure 2I) corroborating the proposition that TGF-β promotes blood vessel maturation.^19^ To verify the permeability of blood vessels, tomato lectin was administered into the tail vein of the mice. In tumor tissues generated in the TβRII^iΔEC^ mice, a greater exudation of lectin was observed from blood vessels when compared with that in the controls, confirming heightened vascular fragility in TβRII^iΔEC^ mice (Figure 2J). In summary, our findings suggest that TGF-β signaling within tumor tissue suppresses vascular EC proliferation and vessel fragility, thereby enhancing vascular maturation.

**Figure 3:**
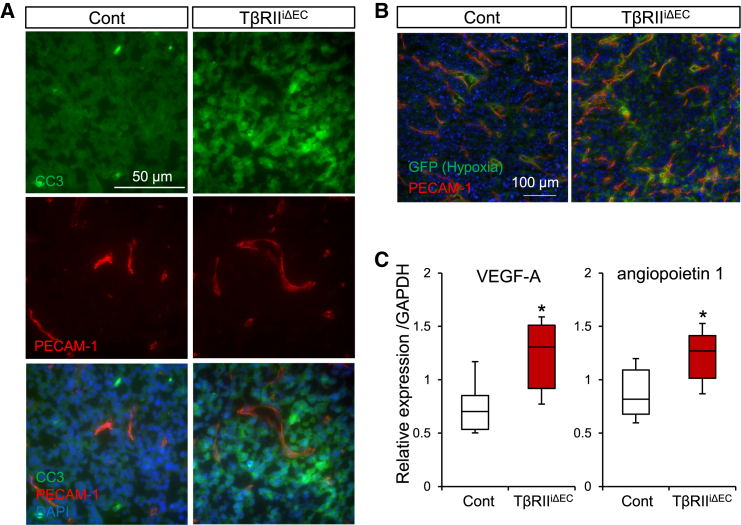
Influence of endothelial TβRII deficiency on angiogenesis and hypoxia induction (A) Immunofluorescence staining of tumor sections with cleaved caspase-3 (CC3) antibodies. TβRII^iΔEC^ mice exhibited increased tumor cell proliferation and apoptosis. (B) Analysis of tumor-bearing mice transplanted with LLC cells expressing GFP in response to hypoxia. Frozen sections of tumor tissues from control and TβRII^iΔEC^ mice were stained with anti-GFP (green) and anti-PECAM-1 (red) antibodies. (C) Quantitative PCR analysis of tumor samples from control and TβRII^iΔEC^ mice. Data are presented as means ± SDs. (unpaired two-tailed t-test, *n* = 6 mice/group ∗*p* < 0.05).

Subsequently, we explored the implications of structural alterations in blood vessels observed in TβRII^iΔEC^ on tumor dynamics. Immunofluorescence analysis of the tumor tissues unveiled an augmented presence of cleaved caspase 3 and Ki-67-positive cells (Figures 3A and 2E), suggesting heightened levels of apoptosis and cell proliferation, respectively. These findings suggest that changes in the vascular architecture may contribute to increased apoptosis and foster cancer cell proliferation, yet tumor growth remained unaltered when compared with that of the control. To investigate the potential of increased fragile blood vessels to impede blood circulation, potentially leading to hypoxia, p5HRE-LLC, expressing enhanced green fluorescent protein (eGFP) in response to a hypoxic environment,^25^ was subcutaneously transplanted into murine backs. Frozen sections obtained 12 days post-transplantation exhibited an expected elevation in the number of GFP-positive cells in the tumors of TβRII^iΔEC^ mice, validating an escalation in hypoxia (Figure 3B). To further elucidate the effect of the deletion of TGF-β signaling deletion in tumor ECs, we directly assessed the secretion of established angiogenic factors in these tumor-bearing mice using a mouse angiogenesis antibody array (Figure S3) in conjunction with subsequent real-time quantitative PCR (qPCR), which revealed elevated relative expressions of proangiogenic factors, including VEGF-A and angiopoietin 1, in the TβRII^iΔEC^ mice when compared with those in the control mice (Figure 3C). Notably, these proangiogenic factors recognized for their role in promoting EC proliferation, were implicated in the heightened tumor angiogenesis observed in TβRII^iΔEC^ mice.

### Reduction in pulmonary metastasis despite an increase in circulating tumor cells in transforming growth factor β type II receptor^iΔEC^ mice

**Figure 4:**
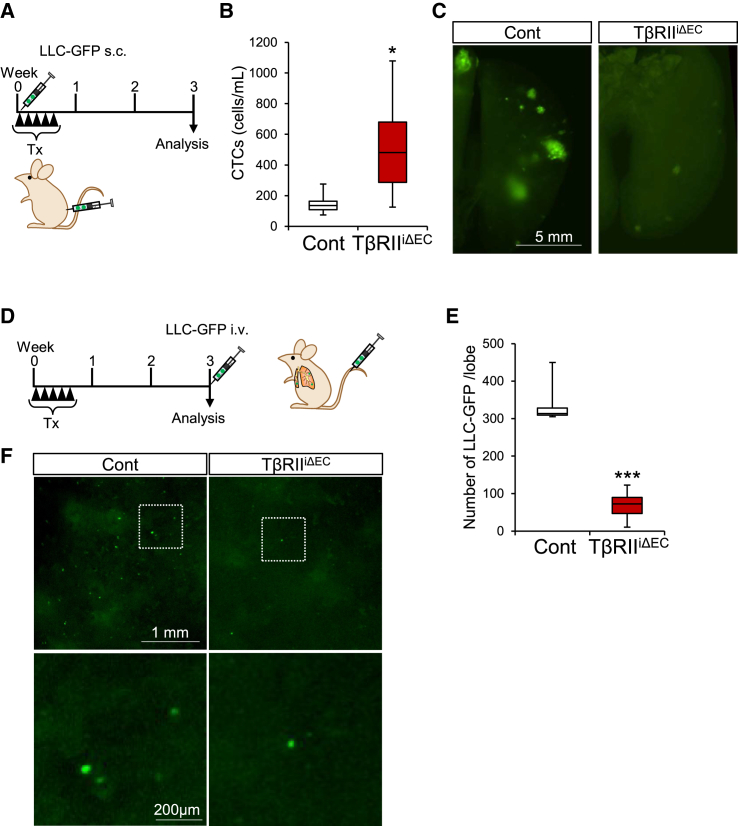
Attenuated pulmonary metastasis despite enhanced circulating tumor cells (CTCs) in TβRII^iΔEC^ mice (A) Experimental timeline depicting the protocol for the spontaneous metastasis model transplanted with LLC-GFP cells. (B) Flow cytometry analysis of CTCs 3 weeks post-transplantation of LLC-GFP cells. CTCs were significantly elevated in the TβRII^iΔEC^ mice when compared with the controls. Data are presented as means ± SDs. (unpaired two-tailed t-test, *n* = 6 mice/group, ∗*p* < 0.05). (C) Representative lung images depicting tumor metastasis 3 weeks after LLC-GFP transplantation. (D) Schedule for the experimental metastasis model using LLC-GFP cells administered via tail vein injection (i.v.). (E and F) Counting of LLC-GFP cells engrafted in the lungs 4 h after i.v. administration. The number of metastatic LLC-GFP cells was compared (E). Data are presented as means ± SDs. (unpaired two-tailed t-test, *n* = 6 mice/group, ∗∗∗*p* < 0.001). Representative image of engrafted cells in a left lung lobe is shown in (F).

Next, we probed the repercussions of alterations in the cancer microenvironment observed in TβRII^iΔEC^ mice on cancer cell metastasis, focusing on the identification of CTCs, which are cancer cells that leave the primary tumors and enter the circulation. Subcutaneous implantation of LLCs stably expressing eGFP (LLC-GFP) was performed in both the control and the TβRII^iΔEC^ mice. LLC-GFP cells show no difference in tumorigenicity compared to LLC and p5HRE-EGFP (HRP) cells (Figure S4). Tamoxifen was administered immediately after implantation for 5 consecutive days (Figure 4A). Three weeks later, blood samples were obtained from these mice, and GFP-positive cells in the peripheral blood were quantified through FACS analysis (Figure 4B). The number of CTCs in the TβRII^iΔEC^ mice exhibited a significant increase when compared with that in the control mice (Figure 4B). Interestingly, despite the elevated number of CTCs in TβRII^iΔEC^ mice, the number of metastasized foci adhering to lung ECs was decreased (Figure 4C).

During the process of tumor metastasis, cancer cells must pass through a vascular endothelial barrier at least twice: first when they escape from the primary tumor and then when they engraft at the metastatic site. Upon arrival at the metastatic site via the bloodstream, cancer cells initially adhere to vascular ECs. In our result, although endothelial cell-specific TβRII deficiency promoted intravascular invasion of cancer cells from the primary tumor, it paradoxically suppressed lung metastasis. The suppression of metastatic foci formation in the lungs may be explained by the hypothesis that cancer cells face impediments in engrafting at lung blood vessels of TβRII^iΔEC^ mice. To investigate this paradox, LLC-GFP cells were introduced via the tail vein in both the control and the TβRII^iΔEC^ mice (Figure 4D). Four hours later, the count of cancer cells engrafted in the lungs was assessed. Before lung removal, PBS perfusion was conducted for removal of all LLC-GFP cells from the bloodstream, ensuring subsequent transparency of the lungs for accurate measurements. The findings showed that LLC-GFP engrafted in the lungs was significantly reduced in the TβRII^iΔEC^ mice (Figures 4E and 4F).

### Suppression of pulmonary metastatic progression in transforming growth factor β type II receptor^iΔEC^ mice

**Figure 5:**
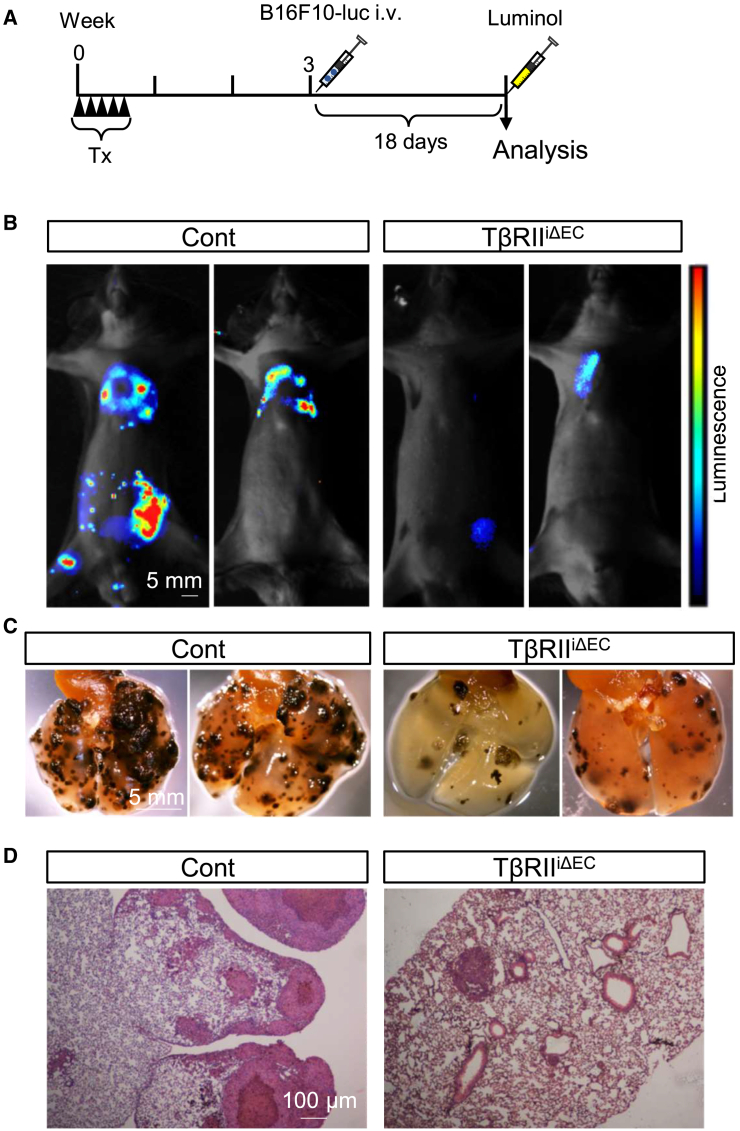
Suppression of tumor metastasis by endothelial TβRII deficiency (A) Schematic representation of the experimental design for the metastasis model employing B16-F10-luc melanoma cells administered intravenously. (B) Representative image displaying the luciferase signal emitted by mice injected with B16F10-luc cells. Tumor progression was assessed by detecting the luciferase signal by use of *in vivo* optical imaging on days 18 postinjection. (C) Representative photographic images of lungs of the control and TβRII^iΔEC^ mice on day 18. (D) Representative histopathologic lung sections obtained on day 18. The sections were stained with hematoxylin and eosin (HE) for visualization. Scale bars: 100 μm.

Following the investigation with LLC-GFP cells, we extended our metastasis experiments to include B16F10 melanoma cells, aiming to explore the broader implications of endothelial TβRII deficiency on metastatic processes. B16F10 melanoma cells expressing luciferase (B16F10-luc cells) were injected via the tail vein 3 weeks after tamoxifen was administered for 5 successive days (Figure 5A). Bioluminescence imaging was performed 18 days after the injection of luciferase-expressing cancer cells (Figure 5B). Metastasized cells were detected in regions beyond the lungs due to the intravenous injection route. However, in the TβRII^iΔEC^ mice, the bioluminescence signals were reduced or undetectable compared to those in the control mice, indicating reduced metastasis in the TβRII^iΔEC^ mice. When the lungs were removed from both groups of mice and the metastasized B16F10-luc cells were examined, more metastasized B16F10-luc cells were observed in the control mice than in the TβRII^iΔEC^ mice (Figure 5D). This experimental result in tumor metastasis model aligns with the findings presented in Figure 4. Once again, when TβRII is specifically deficient in vascular ECs, it becomes challenging for cancer cells to engraft, even when the same amount of circulating cancer cells is present.

### CD44 expression is induced by transforming growth factor β type II receptor, facilitating enhanced adhesion between endothelial cells and Lewis lung carcinoma cells

**Figure 6:**
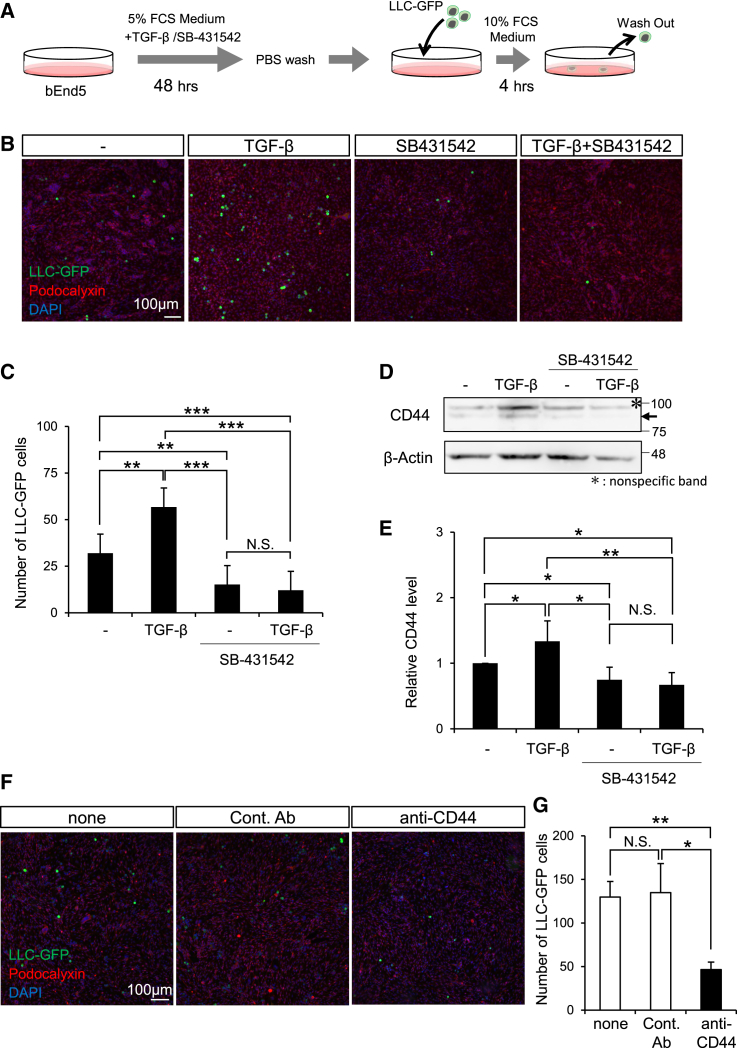
Influence of TGF-β on CD44 expression and cancer cell adhesion to ECs (A) Experimental schedule for adhesion assay between LLC and bEnd5 cells. (B and C) Adhesion analysis of bEnd5 endothelial cells and LLC-GFP cells under TGF-β signaling. bEnd5 cells were cultured for 2 days with the addition of TGF-β (5 ng/mL), the ALK5 inhibitor SB-431542 (10 μmol/mL), or a combination of TGF-β and SB-431542. LLC-GFP cells were then introduced, and after 4 h of coculture, the cells were examined by use of a fluorescence microscope (B) to quantify the number of cancer cells adherent to vascular endothelial cells per unit area (C). Data are presented as means ± SDs. (ordinary 1-way ANOVA, *n* = 5 represents independent experiments, ∗*p* < 0.05; ∗∗*p* < 0.01; ∗∗∗*p* < 0.001; N.S., not significant). (D) Modulation of CD44 expression on bEnd5 cells by TGF-β signaling. After treatment with TGF-β (5 ng/mL), ALK5 inhibitor SB-431542 (10 μmol/L) or a combination of TGF-β and SB-431542 for 2 days, CD44 expression on bEnd5 cells was assessed via Western blot analysis. (E) Quantitative analysis of CD44 expression levels. On the basis of the obtained results shown in panel D. Data are presented as means ± SDs. The relative expression levels of CD44 from 5 different experiments were quantified. (ordinary 1-way ANOVA, *n* = 5, ∗*p* < 0.05; ∗∗*p* < 0.01; N.S.: not significant). (F and G) Impact of anti-CD44 antibody treatment on cancer cell engraftment. bEnd5 cells were treated with either control IgG antibody (Cont Ab) or anti-CD44 antibody. Four hours after the addition of LLC-GFP cells, engrafted cancer cells were examined by use of a fluorescence microscope (G) to determine the number of cancer cells adhering to vascular endothelial cells per unit area (F). Data are presented as means ± SDs. (ordinary 1-way ANOVA, *n* = 5 represents independent experiments, ∗*p* < 0.05; ∗∗*p* < 0.01; N.S.: not significant).

We explored the potential influence of TGF-β signal inhibition resulting from endothelial cell-specific TβRII deficiency on the adhesion process between cancer cells and ECs. Mouse brain vascular ECs, bEnd5, were cultured for 2 days in the presence of either TGF-β, the TGF-β type I receptor kinase inhibitor SB-431542, or a combination of TGF-β with SB-431542. Subsequently, LLC-GFP cells were seeded onto bEnd5 cells, and 4 h postseeding (Figure 6A), the adhesion of LLC-GFP to bEnd5 cells was quantified (Figures 6B and 6C). Similarly, B16F10 cells were also used in the same experimental setup to assess whether the observed effects were consistent across different cancer cell types (Figure S5). The findings showed that TGF-β treatment promoted adhesion between both LLC-GFP and B16F10 cells to vascular ECs, whereas treatment with inhibitor alone, or in combination with TGF-β, suppressed this adhesion.

In the metastatic cascade, neoplastic cells undergo dissociation from the primary metastatic lesion, traverse the circulatory system, and ultimately adhere to the vascular ECs within a distant organ. Focusing on this adhesion process, we aimed to identify intercellular adhesion molecules, which are TGF-β targets, involved in the interaction between cancer cells and ECs. Consequently, as reported for cancer cells,^20^ we observed an upregulation of CD44 expression following TGF-β stimulation, while a downregulation of CD44 expression was seen with inhibitor treatment alone or in combination with TGF-β (Figures 6D and 6E). CD44 is a known poor prognostic factor expressed in cancer cells,^27^ promoting the invasion and metastasis of cancer cells through hyaluronic acid. To elucidate the role of CD44 in the adhesion between ECs and cancer cells, we introduced an anti-CD44 antibody into the medium of the ECs and seeded them with LLC-GFP after 1 h. The CD44 antibody significantly suppressed the engraftment of cancer cells onto ECs (Figures 6F and 6G). These findings suggest that CD44 on ECs, upregulated by TGF-β, may play a crucial role in promoting cancer cell engraftment. Interestingly, even in TβRII^iΔEC^ mice transplanted with LLC-GFP, decreased expression of CD44 was observed in pulmonary vascular ECs one month after tamoxifen administration (Figure S6), suggesting that TGF-β signaling regulates CD44 expression independently of the presence of cancer cells.

**Figure 7:**
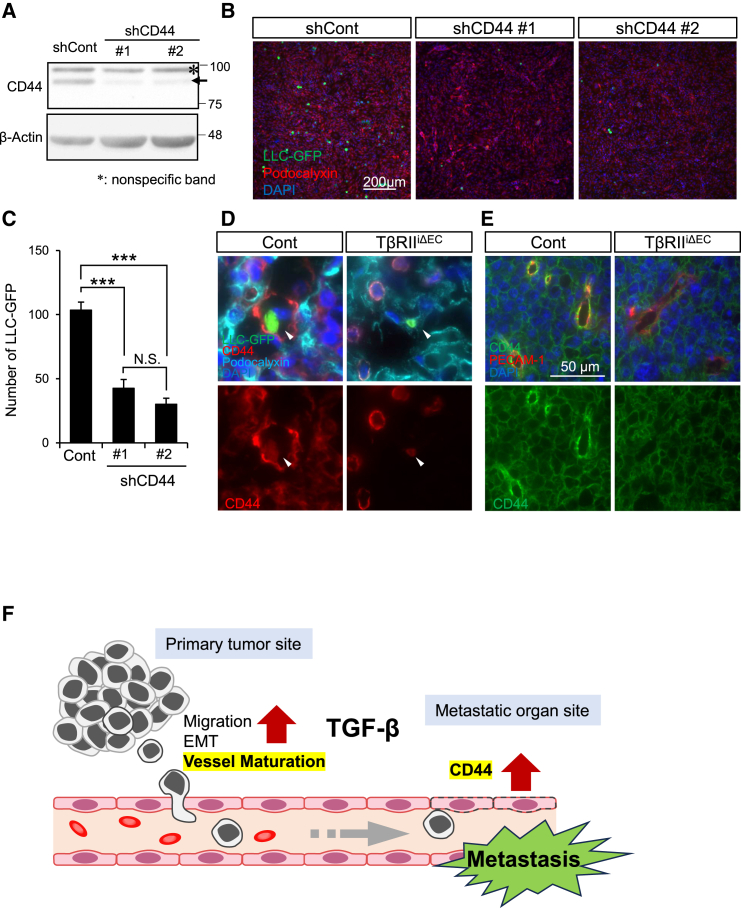
Role of CD44 at ECs in mediating adhesion of cancer cells (A) Western Blot analysis of lentivirus-mediated CD44 knockdown in bEnd5 cells. β-actin served as the loading control. (B and C) Adhesion analysis of bEnd5 cells with CD44 knockdown and LLC-GFP cells. After 4 h of coculture, the adhesion between bEnd5 endothelial cells with CD44 knockdown and LLC-GFP cells was evaluated by use of a fluorescence microscope (B), allowing for the quantification of the number of cancer cells adhering to vascular endothelial cells per unit area (C). Data are presented as means ± SDs. (ordinary 1-way ANOVA, *n* = 3 represents independent experiments, ∗∗∗*p* < 0.001; N.S.: not significant). (D) Immunofluorescence analysis of CD44 expression in lung ECs. Lung sections from the experimental metastasis model mice, as described in Figure 4F, were stained with anti-CD44 antibody (red) and anti-podocalyxin antibody (cyan). Decreased CD44-positive areas were notably observed in the pulmonary vessels of TβRIIiΔEC mice. (E) Tumor sections from the same experiment as in Figure 2 were stained with anti-CD44 antibody (green) and anti-PECAM-1 antibody (red). (F) Illustration of the functional role of TGF-β on tumor metastasis.

To probe the involvement of CD44 in vascular ECs in cancer cell engraftment, bEnd5 cells were infected with scrambled short hairpin RNA (shRNA) and shRNA targeting mCD44 (shCD44). Analysis of CD44 expression revealed a significant decrease in shCD44 #1 and #2, confirming the efficacy of the CD44 suppression (Figure 7A). Subsequently, 4 h postseeding these cells with LLC-GFP, we quantified the number of cancer cells engrafted on these bEnd5 cells. The results clearly demonstrated that the reduction of CD44 expression in vascular ECs significantly diminishes the ability of cancer cells to adhere (Figures 7B and 7C). Furthermore, we extended our investigation to determine the role of CD44 in cancer cell engraftment to vascular ECs in an *in vivo* mouse model. Employing the experimental metastasis model depicted in Figure 4D, frozen lung sections were prepared. Using the fluorescent immunostaining of CD44 at the sites of cancer cell engraftment in vascular ECs, we observed a robust positivity for CD44 in the regions where cancer cells had adhered (Figure 7D), as illustrated in Figure 7G. Additionally, fluorescent immunostaining images of CD44 expression from frozen tumor tissue sections in Figure 2 revealed reduced CD44 expression in the blood vessels within the tumor tissue (Figure 7E). These findings provide compelling evidence that some cancer cells indeed engraft ECs via CD44, even in the context of cancer metastasis within living organisms (Figure 7F).

## Discussion

Tumor angiogenesis is a critical process in cancer progression, facilitating the establishment of new blood vessels that nourish the tumor, promote cell proliferation, and foster metastasis.^2^ This vascular network not only sustains the primary tumor but also serves as a conduit for cancer cells to infiltrate the bloodstream, facilitating dissemination and profoundly shaping the tumor microenvironment.^14^^,^^28^ During late-stage tumorigenesis, heightened production of TGF-β by cancer cells assumes a pivotal role in driving progression by fostering an immunosuppressive milieu, fueling angiogenesis, and amplifying the invasive potential through mechanisms such as the EMT.^12^^,^^13^ TGF-β exhibits a dualistic character in angiogenesis,^17^^,^^29^ simultaneously stimulating the production of proangiogenic factors and exerting antiangiogenic effects. Despite this dual role, elucidation of the precise effects of TGF-β on the angiogenic switch within the tumor microenvironment has revealed a nuanced regulation of tumor angiogenesis at the level of ECs. Selective inhibition of TGF-β signaling in vascular ECs through the targeted deletion of the *TβRII* gene, under the control of the Pdgfb promoter, led to an unexpected activation of tumor angiogenesis (Figure 2). This effect stemmed not only from the alleviation of TGF-β signal-induced growth inhibition (Figure 2E) but also from an augmented expression of angiocrine factors linked to alteration in the cancer microenvironment (Figure 3C). Our study elucidates the intricate role of TGF-β signaling in tumor angiogenesis and metastasis, unveiling unexpected findings that challenge conventional perspectives. Whilst TGF-β has long been recognized for its multifunctional roles in cancer progression, our investigation reveals a previously unreported mechanism of tumor angiogenesis at the level of ECs. This finding underscores the complexity of TGF-β signaling dynamics and its profound influence on the tumor microenvironment. This finding underscores the complexity of TGF-β signaling dynamics and its profound influence on the tumor microenvironment.

Inhibitors targeting TGF-β signaling have shown efficacy in reducing tumor growth and metastasis.^30^^,^^31^ Whilst these inhibitors primarily target cancer cells, our observations suggest that the inhibition of TGF-β signaling in ECs could influence tumor vessels. Tumor blood vessels formed in TβRII^iΔEC^ mice exhibited extreme fragility, leading to recurrent bleeding (Figures 1E, 2I, and 2J). This fragility stems from the role of TGF-β signaling in blood vessel maturation,^19^^,^^32^ where the process of newly established vessel maturation is impaired. These results align with observations of fragile vessels of TβRII^iΔEC^ neonatal retinas (Figure S7).^17^^,^^33^^,^^34^ Moreover, it is noteworthy that hypoxia, a common feature of the tumor microenvironment, can enhance TGF-β signals,^35^ thereby exacerbating the effects observed in our study. These fragile blood vessels create a hypoxic tumor microenvironment, triggering both apoptosis and proliferation of cancer cells, culminating in overall tumor growth comparable to that in controls. Although overall tumor size was equivalent, the increased number of circulating tumor cells (CTCs) suggests that the inhibition of TGF-β in ECs may provide cancer cells with greater opportunities for metastasis. Our results show the delicate balance between the formation of angiogenesis and metastasis, suggesting that treatments targeting TGF-β signaling need to be approached with caution.

Previous studies have reported the complex role of TGF-β signaling in cancer progression and angiogenesis, particularly its regulation of angiogenic factors such as VEGF-A and angiopoietin-1.^11^^,^^12^ In epithelial ovarian cancer and colorectal cancer, TGF-β was shown to contribute to angiogenesis through the upregulation of VEGF-A and other pro-angiogenic factors.^36^^,^^37^ In peritoneal carcinomatosis of colorectal cancer, the tumor microenvironment undergoes significant structural and functional reorganization. This reorganization includes hypoxia-induced changes that interact with TGF-β signaling, contributing to the modulation of angiogenic factors and influencing tumor progression through processes such as cellular senescence and proliferation arrest.^38^ While these studies provide insights into TGF-β′s role in cancer cells and the tumor microenvironment, we specifically examined if the loss of TβRII affected ECs. In contrast to the pro-angiogenic effects of TGF-β in tumor cells, we observed that loss of TGF-β signaling in ECs led to increased expression of VEGFA and angiopoietin-1, suggesting distinct mechanisms within the endothelial compartment. This endothelial-specific response may contribute to the abnormal angiogenesis and compromised vascular integrity observed in our model. These findings emphasize the multifaceted nature of TGF-β signaling in tumor angiogenesis and highlight the importance of considering cell-type-specific effects when interpreting its role in cancer progression.

These findings emphasize the multifaceted nature of TGF-β signaling in tumor angiogenesis and highlight the importance of considering cell-type-specific effects when interpreting its role in cancer progression. Notably, EC-specific inhibition of TGF-β signaling influenced metastasis. Whilst TβRII^iΔEC^ mice exhibited an augmented presence of CTCs, lung metastasis was significantly reduced. This paradox may be explained by the inability of cancer cells to engage ECs in the metastatic niche or by alterations in the tumor microenvironment that hinder the escape of cancer cells from the primary tumor. However, as most CTCs exist as single cells with a short half-life in circulation,^39^^,^^40^ the latter hypothesis in the tumor microenvironment is hindering the escape of cancer cells from the primary tumor seems more likely. Indeed, experiments using the LLC-GFP metastasis model in control and TβRII^iΔEC^ mice demonstrated a reduced capacity for lung vascular ECs to harbor cancer cells post-gene deletion. Recent studies have emphasized the inclination of cancer cells to aggregate into clusters and employ platelet shields as a means to evade immune surveillance. α-granules within platelets work as a substantial reservoir of TGF-β, which is released upon platelet activation.^41^^,^^42^ Platelet-derived TGF-β can trigger TGF-β/Smad and NF-κB pathways in tumor cells, prompting an invasive mesenchymal-like phenotype that enhances the metastatic potential. Therefore, the inhibition of TGF-β or NF-κB signaling could offer a promising therapeutic strategy to reduce lung metastasis.^43^

Despite the theoretical potential of clustered cells to impede capillary blood flows and promote metastasis, individual cancer cells must adhere to the ECs of the metastatic organ to establish successful metastasis. Cell-cell adhesion is crucial for cancer cells to anchor onto endothelial cells (ECs) despite the force of blood flow. Various types of leukocytes, for instance, traverse ECs by rolling through intercellular adhesion factors.^22^^,^^44^ In the context of cancer metastasis observed in TβRII^iΔEC^ mice, cancer cells may activate the signaling pathway through CD44, a well-known cancer stem marker,^45^^,^^46^ whose expression is increased by TGF-β signaling.^20^ CD44 is involved in cell-cell interactions and migration,^47^ and also activates MMP-9 which promotes tumor angiogenesis through TGF-β activation. Blockage of CD44 signal with an antibody not only inhibited hematopoietic stem cell migration^48^ but also suppressed lung metastases in cancer models (Figure 6). CD44 acts as a receptor for hyaluronic acid, and previous studies indicate that CD44 expressed on both ECs and cancer cells engages in cell-cell adhesion through a sandwich structure involving hyaluronic acid.^47^^,^^49^ Our study revealed an association between decreased TGF-β signaling and reduced CD44 expression *in vitro* and *in vivo* (Figure 6): as well as the utilization of the conventional CD44 expressed by LLC and mouse vascular ECs, the addition of an anti-CD44 antibody or CD44 knockdown successfully inhibited the engraftment of cancer cells onto ECs (Figure 7). By elucidating the involvement of CD44 in facilitating cancer cell engraftment onto vascular ECs, we have here unveiled potential therapeutic avenues for disrupting this process. However, further investigation into the impact of CD44 splicing variants on cancer cell engraftment is warranted, given their reported association with poor prognosis in patients with cancer .

In conclusion, our study sheds light on the nuanced role of TGF-β signaling in tumor angiogenesis and metastasis. The observed negative regulation of tumor angiogenesis at the level of ECs challenges conventional perspectives on TGF-β′s function in cancer progression. Moreover, our findings underscore the intricate balance required in targeting TGF-β signaling, exemplified by the fragility of tumor blood vessels and their consequential impact on the tumor microenvironment. Notably, the inhibition of TGF-β signaling in ECs significantly perturbs tumor vessels, highlighting the intricate interplay between angiogenesis and metastasis. Furthermore, our insights into CD44-mediated cell-cell adhesion unveil potential therapeutic avenues, warranting further investigation on cancer engraftment. Overall, these findings deepen our comprehension of the molecular intricacies underlying cancer metastasis and offer a promising direction for targeted interventions with broader therapeutic implications.

### Limitations of the study

Limitations of our study include the use of transplanted tumor models (Lewis lung carcinoma and B16F10 melanoma), which do not fully replicate the complexity of naturally occurring cancers. Spontaneously arising tumors *in vivo* exhibit clonal heterogeneity, which can influence responses to TGF-β signaling and CD44-mediated adhesion, potentially affecting the generalizability of our findings. Additionally, while we observed reduced lung metastasis associated with CD44 downregulation, further investigation into the role of different CD44 splicing variants across various cancer types would provide a more comprehensive understanding of TGF-β′s impact on metastasis.

## Resource availability

### Lead contact

Further information and requests for resources and reagents should be directed to and will be fulfilled by the Lead Contact, Dr. Fumiko Itoh (mame.fumiko@gmail.com).

### Materials availability

Plasmids and cell lines generated in this study are available from lead contact under a material transfer agreement with the Tokyo University of Pharmacy and Life Sciences.

### Data and code availability

• Data: All data reported in this article will be shared by the lead contact upon request.
• Code: This article does not report any code.
• Other items: Any additional information required to reanalyze the data reported in this article is available from the lead contact upon request

## Acknowledgments

This research was supported by the 10.13039/501100001700Japanese Ministry of Education, Culture, Sports, Science, and Technology (23KJ1956 to K.H. and 23K06488 to F.I.). We would like to thank Ms F. Miyamasu for English language editing and Mr. T. Yamashita for scientific support.

## Author contributions

Conceptualization, F.I.; methodology, K.H., T.T., and S.I.; validation, K.H., Y.S., T.T., G.M., and S.I.; investigation, K.H., Y.S., T.T., M.G., Y.N., T.I., and F.I.; writing – original draft, F.I; writing – review and editing, M.F., S.I, and F.I.; funding acquisition, K.H. and F.I.; resources, H.H., M.F., and S.I.; project administration, F.I.; supervision, F.I.

## Declaration of interests

The authors have no conflicts of interest.

## STAR★Methods

### Key resources table

**Table:** 

REAGENT or RESOURCE	SOURCE	IDENTIFIER
**Antibodies**		

Rat monoclonal anti-PECAM-1	BD Biosciences	Cat# 550274; RRID: AB_393571
Goat anti VEGFR3	R&D systems	Cat# AF743; RRID: AB_355563
Mouse anti-Actin, alpha-Smooth Muscle – Cy3 ™	Millipore Sigma	Cat# C6198; RRID: AB_476856
α-Smooth Muscle Actin Rabbit	Cell Signaling Technology	Cat# 19245; RRID: AB_2734735
Rat anti-mouse TER119/Erythroid Cells	BD Pharmingen™	Cat# 561033; RRID: AB_10584336
Rabbit polyclonal pSmad2	homemade	N/A
Anti-TGF beta Receptor II	abcam	Cat# ab184948; RRID: AB_2818975
Anti-Ki67 antibody [SP6]	abcam	Cat# ab16667; RRID: AB_302459
Cleaved Caspase-3 (Asp175)	Cell Signaling Technology	Cat# 9661; RRID: AB_2341188
Monoclonal Anti-β-Actin, Clone AC-74	Sigma-Aldrich	Cat# A2228; RRID: AB_476697
Purified anti-mouse/human CD44	BioLegend	Cat# 103001; RRID: AB_312952
Alexa 488-conjugated donkey anti-rabbit IgG	Thermo Fisher Scientific	Cat# A-21206; RRID: AB_2535792
Alexa 488-conjugated goat anti-rabbit IgG	Thermo Fisher Scientific	Cat# A-11008; RRID: AB_143165
Alexa 594-conjugated goat anti-rat IgG	Thermo Fisher Scientific	Cat# A-21208; RRID: AB_2535794
DAPI	DOJINDO LABORATORIES	D523
PE anti-mouse CD309 (VEGFR2, Flk-1)	BioLegend	Cat# 136403; RRID: AB_1967093
ECL™ Anti-mouse IgG, Horseradish Peroxidase-linked Whole Antibody (from sheep), 1mL	GE Healthcare Life Sciences	Cat# NA931; RRID: AB_772210
ECL™ Anti-rat IgG, Horseradish Peroxidase-linked Whole Antibody (from goat), 1mL	GE Healthcare Life Sciences	Cat# NA935; RRID: AB_772207

**Bacterial and virus strains**		

TG1 Chemically Competent E.Coli	Our Lab	N/A
pLKO.1^53^-shControl	This paper	N/A
pLKO.1^53^-shCD44	This paper	N/A

**Chemicals, peptides, and recombinant proteins**		

Tamoxifen	Sigma-Aldrich	T5648
TGF-β1	R&D Systems	240-B
SB-431542	Selleck Chemicals	301836-41-9
TRIzol™ Reagent	Thermo scientific	15596026
FBS	Biological Industries Ltd.	04-001-1A
DMEM	Nacalai Tesque	08458–16
MEM Nonessential Amino Acids Solution	Nacalai Tesque	06344–58
penicillin/streptomycin	FUJIFILM Wako Pure Chemical Corporation	168–23191
hygromycin B	FUJIFILM Wako Pure Chemical Corporation	089–06151
Zeocin	Invitrogen	R25001
polybrene	Sigma-Aldrich	107689

**Critical commercial assays**		

PrimeScript II™ 1st strand cDNA Synthesis Kit	Takara Bio Inc	6210A
Mouse Angiogenesis Antibody Array	R&D Systems	# ARY015
Super Signal West Dura Extended Duration Substrate	Thermo scientific	34076
NucleoBond*®* Xtra Midi	Takara Bio Inc	U0410C
KAPA SYBR FAST qPCR Kit	NIPPON Genetics	KK4611
KAPA SYBR FAST qPCR Kit	NIPPON Genetics	KK4611

**Experimental models: Cell lines**		

LLC	ATCC	CLR1642
B16F10	ATCC	CRL-6475
bEnd5	Dr. Deutsh U.^50^	N/A
LLC-p5HRE	Our Lab.	N/A
LLC-GFP	Our Lab.	N/A

**Experimental models: Organisms/strains**		

TβRII^fl/fl^ mice	Dr. Karlsson S.^51^	N/A
Pdgfb-iCreER mice	Dr. Fruttiger M.^25^	N/A
TβRII^fl/fl;^Pdgfb-iCreER mice on a C57BL/6J background	This Paper	N/A

**Oligonucleotides**		

shRNA targeting sequence: shCD44#1: 5′- ccggAATTCCTTCGATGGACCGGTT ctcgagAACCGGTCCATCGAAGGAATTtttttg-3′ and 5′- aattcaaaaaAATTCCTTCGATGGACCGGT TctcgagAACCGGTCCATCGAAGGAATT-3′	This paper	N/A
shRNA targeting sequence: shCD44#2: 5′- ccggAAGTGACTCTTGCTACTGACTct cgagAGTCAGTAGCAAGAGTCACTTtttttg and 5′- aattcaaaaaAAGTGACTCTTGCTACTGACT ctcgagAGTCAGTAGCAAGAGTCACTT −3	This paper	N/A
shRNA targeting sequence: shControl: 5′- ccggCCTAAGGTTAAGTCGCCCTCGctc gagCGAGGGCGACTTAACCTTAGGtttttg-3′ and 5′-aattcaaaaaCCTAAGGTTAAGTCGCCCTC GctcgagCGAGGGCGACTTAACCGGAGG-3′	This paper	N/A
Primers for qRT-PCR, see Table S2	This paper	N/A

**Recombinant DNA**		

pGL4.50 [luc2/CMV/hygro] vector		

**Software and algorithms**		

BZ-X800 Analyzer	KEYENCE CORPORATION	N/A
ImageJ	NIH ImageJ	https://imagej.net/ij/
Adobe Photoshop	Adobe	https://www.adobe.com/products/photoshop.html#
LightCycler*®* 96	Roche	N/A
DNA Dynamo	Blue Tractor Software	https://www.bluetractorsoftware.com/

**Other**		

Keyence VHX-2000 zoom microscope	KEYENCE CORPORATION	N/A
BZ-X810	KEYENCE CORPORATION	N/A

### Experimental model and study participant details

#### Mice

TβRII^fl/fl^ and Pdgfb-iCreER mice, maintained on a C57BL/6J background, have been previously described.^25^^,^^51^ Tamoxifen (Tx) (T5648; Sigma-Aldrich, St. Louis) dissolved in corn oil (20 mg/mL), was intraperitoneally administered (40 mg/kg/day) for 5 consecutive days into control (TβRII^fl/fl^) and TβRII^fl/fl^; Pdgfb-icreER mice, in which the *TβRII* gene in vascular ECs (ECs) is deficient (TβRII^iΔEC^). All the animal experiments were approved by the animal study committee (Tokyo University of Pharmacy and Life Sciences) and performed in accordance with the Guidelines of the Tokyo University of Pharmacy and Life Sciences for Animal and Recombinant DNA Experiments (L20–5, L21–13, L22–16, L23-15). The mice were housed in the animal facilities of Tokyo University of Pharmacy and Life Sciences and Showa Pharmaceutical University under specific pathogen-free conditions with constant temperature and humidity and were provided a standard diet.

#### Cell line and cell culture

LLC (PRID:CLR1642) and B16F10 (PRID: CRL-6475) cells obtained from ATCC were cultured in Dulbecco’s modified Eagle’s medium (DMEM; Nacalai Tesque, Kyoto, Japan) containing 10% fetal bovine serum (FBS; Biological Industries Ltd., Israel), 1 x MEM nonessential amino acids (Nacalai Tesque) and 100 U/mL penicillin/streptomycin (PS; Wako, Osaka, Japan). The culture of bEnd5 cells^50^ was additionally supplemented with 0.15 mM β-mercaptoethanol. LLC-cells carrying p5HRE-EGFP or eGFP (LLC-GFP) were maintained with 1200 μg/mL hygromycin B (Wako, Tokyo, Japan) or 600 mg/mL Zeocin (Invitrogen), respectively. B16-F10 cells carrying the pGL4.50 [luc2/CMV/hygro] vector were maintained with 1200 μg/mL hygromycin B. bEnd5 cells expressing the constructs indicated in each figure were isolated using a retrovirus expression system and maintained in puromycin (2 μg/mL)-containing medium. The cell lines used in this study were obtained from a certified source and were confirmed to be mycoplasma-free.

### Method details

#### Xenograft model

LLC, LLC-p5HRE, or LLC-GFP cells (2.5 × 10^5^ cells/100 μL PBS) were transplanted slightly to the right, about 1 cm from the base of the tail to the back of male TβRII^fl/fl^ and TβRII^iΔEC^ mice (7–12 weeks old). Tumor volumes (V) were calculated by use of the following formula: 0.5 × length × width × width.^26^ The tumors were surgically removed and embedded into a frozen section compound (Leica) for further immunofluorescence analysis. To detect spontaneous metastasis, the lungs were removed from the mice 3 weeks after LLC-GFP transplantation and exudation in CUBIC reagent.^26^ To measure CTCs, 1 mL of blood from tumor-bearing TβRII^fl/fl^ and TβRII^iΔEC^ mice was collected 3 weeks after transplantation of LLC-GFP cells. The blood was then incubated with 20 mL of ACK buffer (0.15 M NH_4_Cl, 1 mM KHCO_3_, and 0.1 mM EDTA) to remove the red blood cells. After being washed with PBS containing 2% FBS, the cells were analyzed by use of a CytoFLEX flow cytometer (Beckman Coulter). Absolute CTC values were determined by use of a standard flow counting fluorosphere (Beckman Coulter). The study was not blinded. Luciferase-expressing B16-F10 cells (4 × 10^4^ cells) were intravenously injected into TβRII^fl/fl^ and TβRII^iΔEC^ mice after 3 weeks of Tx administration. At the indicated dates, the anesthetized mice were intraperitoneally administered with 150 mg/kg d-luciferin potassium salt (Wako). Subsequently, bioluminescence in each mouse was monitored using an ImagEM C9100 EM-CCD camera (Hamamatsu Photonics) and AQUA COSMOS imaging software (Hamamatsu Photonics). The study was not blinded. The mice were treated in accordance with the institutional guidelines of the Animal Care and Use Program of Showa Pharmaceutical University (approval P-2024-5, dated April 15, 2024).

#### Immunofluorescence analysis and quantification

The antibodies we used are shown in Table S1. Fresh frozen sections (5 μm) were cut with a CM1850 cryostat (Leica Camera AG, Wetzlar, Germany), mounted on Cryofilm (Leica), and fixed in 100% ethanol and 4% PFA. The films were washed three times with PBS, permeabilized with 0.1% Triton X-100 (Sigma) for 5 min, and blocked with Blocking Reagent (Dako, Glostrup, Denmark) for 1 h at 37°C. First antibodies in Blocking Reagent were added and incubated overnight at 4°C. The films were washed three times with PBS and then incubated with secondary antibodies. After the nuclei as needed were stained with 2 μg/mL DAPI (Dojindo Laboratories, Kumamoto, Japan) for 5 min, the samples were washed three times with PBS, and the fluorescence signals were visualized by BZ-X 810 fluorescence microscope (Keyence, Osaka, Japan). We quantified using two 10x images per mouse, with three mice each for the TβRII^fl/fl^ and TβRII^iΔEC^ mice.

#### Lung engraftment of cancer cells

LLC-GFP cells (4.0 × 10^4^ cells/200 μL PBS) were intravenously injected into male TβRII^fl/fl^ and TβRII^iΔEC^ mice 3 weeks after Tx administration. After 4 h, PBS was perfused to remove circulating LLC-GFP, and the lungs were excised and clarified in CUBIC. Cancer cells that had engrafted onto ECs in the lungs were identified as GFP-positive cells by use of a stereomicroscope.

#### Lentiviral shRNA

The lentiviral vectors for CD44 shRNA were constructed using a pLKO.1 vector. Lentiviral vectors expressing shCD44 were transfected into 293T cells together with psPAX2 and pMD2.G. After 48 h of transfection, the culture media were collected as a source of lentiviruses.^52^ Subsequently, lentiviruses were simultaneously incubated in DMEM containing 8 μg/mL polybrene (Sigma) for 2 h and then added to the bEnd5 cell culture dishes. Twelve hours after infection, the cells were washed and cultured in medium. Infected bEnd5 cells, which became puromycin-resistant, were used for the experiments. Mouse shCD44#1, shCD44#2, and shControl included the following sequences: and 5′- ccggAATTCCTTCGATGGACCGGTTctcgagAACCGGTCCATCGAAGGAATTtttttg-3′ and 5′- aattcaaaaaAATTCCTTCGATGGACCGGTTctcgagAACCGGTCCATCGAAGGAATT-3’; 5′- ccggAAGTGACTCTTGCTACTGACTctcgagAGTCAGTAGCAAGAGTCACTTtttttg and 5′- aattcaaaaaAAGTGACTCTTGCTACTGACTctcgagAGTCAGTAGCAAGAGTCACTT −3; 5′- ccggCCTAAGGTTAAGTCGCCCTCGctcgagCGAGGGCGACTTAACCTTAGGtttttg-3′ and 5′-aattcaaaaaCCTAAGGTTAAGTCGCCCTCGctcgagCGAGGGCGACTTAACCGGAGG-3’; respectively. Each lentiviral vector was transfected into 293T cells together with psPAX2 and pMD2.G. After 48 h of transfection, the media were collected as a source of lentiviruses. Each lentivirus was incubated in DMEM containing 8 μg/mL polybrene (Sigma) for 2 h and then added to the bEnd5 cell culture dishes. Twelve hours after infection, the cells were washed and cultured in medium. Infected bEnd5 cells, which became puromycin-resistant, were used for the experiments.

#### Western blot analysis

To detect the protein expression, cells were lysed in 500 μL TNE buffer containing 10 mM Tris (pH 7.4), 150 mM NaCl, 1 mM ethylenediamine-N′, N′, N′, N′-tetraacetic acid (EDTA), 1% NP-40, 1 mM phenylmethylsulfonyl-*l*-fluoride (PMSF), 5 μg/mL leupeptin, 100 U/mL aprotinin, 2 mM sodium vanadate, 40 mM NaF, and 20 mM β-glycerophosphate. The cell lysates were then boiled for 5 min in sample buffer, separated by means of SDS-polyacrylamide gel electrophoresis (SDS-PAGE), and transferred to an UltraCruz Nitrocellulose Pure Transfer membrane (Santa Cruz Biotechnology, CA). After being transferred, the membranes were probed with primary antibodies. The primary antibodies were subsequently detected using horseradish peroxidase-conjugated secondary antibodies and a chemiluminescent substrate (Thermo Fisher Scientific, Waltham, MA). The chemiluminescence signal was captured using a luminescence image analyzer (WSE-6100 LuminoGraph I, ATTO Corporation, Tokyo, Japan).

#### RNA preparation and quantitative real-time PCR (qPCR) analysis

Total RNA from mouse tissues was extracted by use of TRIzol Reagent (Thermo Fisher Scientific). Reverse transcription was performed with a PrimeScript II 1^st^ strand cDNA Synthesis Kit (Takara Bio Inc). Subsequently, qPCR was carried out using the KAPA SYBR FAST qPCR Master Mix (2X) Kit (Kapa Biosystems, Cape Town, South Africa). All reactions were carried out on a LightCycler 96 (Roche), with each sample analyzed at least twice for each PCR measurement. The primers we used are shown in Table S2. Melting curves were checked to ensure specificity. Relative quantification of mRNA expression was calculated using the standard curve method with the GAPDH or β-actin level as the reference. Before qPCR, the DNA fragment amplified using each primer set was verified to yield a single band of the correct size in agarose gel electrophoresis.

### Quantification and statistical analysis

Results were expressed as means ± standard deviation (SDs). Statistical significance was assessed using one-way analysis of variance (ANOVA), with the Least Significnt Differnce (LSD) method applied for comparison between two groups or an unpaired Student’s t test. *p*-values of ∗*p* < 0.05, ∗∗*p* < 0.01, ∗∗∗*p* < 0.001 were considered significant. N.S. not significant, *p*-value >0.05. The relevant statistical main factors are shown in Table S3. The tests applied to each experiment are indicated in the figure legends.

## References

[1] Folkman J. (1971). Tumor angiogenesis: therapeutic implications. N. Engl. J. Med..

[2] Weis S.M., Cheresh D.A. (2011). Tumor angiogenesis: molecular pathways and therapeutic targets. Nat. Med..

[3] Carmeliet P. (2003). Angiogenesis in health and disease. Nat. Med..

[4] De Palma M., Biziato D., Petrova T.V. (2017). Microenvironmental regulation of tumour angiogenesis. Nat. Rev. Cancer.

[5] Chauhan V.P., Stylianopoulos T., Martin J.D., Popović Z., Chen O., Kamoun W.S., Bawendi M.G., Fukumura D., Jain R.K. (2012). Normalization of tumour blood vessels improves the delivery of nanomedicines in a size-dependent manner. Nat. Nanotechnol..

[6] Choi Y., Jung K. (2023). Normalization of the tumor microenvironment by harnessing vascular and immune modulation to achieve enhanced cancer therapy. Exp. Mol. Med..

[7] Liu S., Ren J., ten Dijke P. (2021). Targeting TGFβ signal transduction for cancer therapy. Signal Transduct. Target. Ther..

[8] Itoh F., Watabe T., Miyazono K. (2014). Roles of TGF-β family signals in the fate determination of pluripotent stem cells. Semin. Cell Dev. Biol..

[9] Miyazono K., Katsuno Y., Koinuma D., Ehata S., Morikawa M. (2018). Intracellular and extracellular TGF-β signaling in cancer: some recent topics. Front. Med..

[10] Morikawa M., Derynck R., Miyazono K. (2016). TGF-β and the TGF-β family: context-dependent roles in cell and tissue physiology. Cold Spring Harbor Perspect. Biol..

[11] Massagué J., Sheppard D. (2023). TGF-β signaling in health and disease. Cell.

[12] Drabsch Y., ten Dijke P. (2012). TGF-β signalling and its role in cancer progression and metastasis. Cancer Metastasis Rev..

[13] Winkler J., Abisoye-Ogunniyan A., Metcalf K.J., Werb Z. (2020). Concepts of extracellular matrix remodelling in tumour progression and metastasis. Nat. Commun..

[14] Batlle E., Massagué J. (2019). Transforming growth factor-β signaling in immunity and cancer. Immunity.

[15] Nixon B.G., Gao S., Wang X., Li M.O. (2023). TGFβ control of immune responses in cancer: a holistic immuno-oncology perspective. Nat. Rev. Immunol..

[16] Tauriello D.V.F., Sancho E., Batlle E. (2022). Overcoming TGFβ-mediated immune evasion in cancer. Nat. Rev. Cancer.

[17] Goumans M.J., ten Dijke P. (2018). TGF-β signaling in control of cardiovascular function. Cold Spring Harbor Perspect. Biol..

[18] Cunha S.I., Pietras K. (2011). ALK1 as an emerging target for antiangiogenic therapy of cancer. Blood.

[19] Itoh F., Itoh S., Adachi T., Ichikawa K., Matsumura Y., Takagi T., Festing M., Watanabe T., Weinstein M., Karlsson S., Kato M. (2012). Smad2/Smad3 in endothelium is indispensable for vascular stability via S1PR1 and N-cadherin expressions. Blood.

[20] Carmeliet P., Jain R.K. (2011). Principles and mechanisms of vessel normalization for cancer and other angiogenic diseases. Nat. Rev. Drug Discov..

[21] Viallard C., Larrivée B. (2017). Tumor angiogenesis and vascular normalization: alternative therapeutic targets. Angiogenesis.

[22] DeGrendele H.C., Estess P., Picker L.J., Siegelman M.H. (1996). CD44 and its ligand hyaluronate mediate rolling under physiologic flow: a novel lymphocyte-endothelial cell primary adhesion pathway. J. Exp. Med..

[23] Ponta H., Sherman L., Herrlich P.A. (2003). CD44: from adhesion molecules to signalling regulators. Nat. Rev. Mol. Cell Biol..

[24] Zöller M. (2011). CD44: can a cancer-initiating cell profit from an abundantly expressed molecule?. Nat. Rev. Cancer.

[25] Claxton S., Kostourou V., Jadeja S., Chambon P., Hodivala-Dilke K., Fruttiger M. (2008). Efficient, inducible Cre-recombinase activation in vascular endothelium. Genesis.

[26] Fukasawa K., Hanada K., Ichikawa K., Hirashima M., Takagi T., Itoh S., Watabe T., Itoh F. (2021). Endothelial-specific depletion of TGF-β signaling affects lymphatic function. Inflamm. Regen..

[27] Derynck R., Turley S.J., Akhurst R.J. (2021). TGFβ biology in cancer progression and immunotherapy. Nat. Rev. Clin. Oncol..

[28] Mikami S., Mizuno R., Kosaka T., Saya H., Oya M., Okada Y. (2015). Expression of TNF-α and CD44 is implicated in poor prognosis, cancer cell invasion, metastasis and resistance to the sunitinib treatment in clear cell renal cell carcinomas. Int. J. Cancer.

[29] Ten Dijke P., Arthur H.M. (2007). Extracellular control of TGFβ signalling in vascular development and disease. Nat. Rev. Mol. Cell Biol..

[30] Ciardiello D., Elez E., Tabernero J., Seoane J. (2020). Clinical development of therapies targeting TGFβ: current knowledge and future perspectives. Ann. Oncol..

[31] Zhang M., Zhang Y.Y., Chen Y., Wang J., Wang Q., Lu H. (2021). TGF-β signaling and resistance to cancer therapy. Front. Cell Dev. Biol..

[32] Aspalter I.M., Gordon E., Dubrac A., Ragab A., Narloch J., Vizán P., Geudens I., Collins R.T., Franco C.A., Abrahams C.L. (2015). Alk1 and Alk5 inhibition by Nrp1 controls vascular sprouting downstream of Notch. Nat. Commun..

[33] Allinson K.R., Lee H.S., Fruttiger M., McCarty J.H., Arthur H.M. (2012). Endothelial expression of TGFβ type II receptor is required to maintain vascular integrity during postnatal development of the central nervous system. PLoS One.

[34] Franco C.A., Blanc J., Parlakian A., Blanco R., Aspalter I.M., Kazakova N., Diguet N., Mylonas E., Gao-Li J., Vaahtokari A. (2013). SRF selectively controls tip cell invasive behavior in angiogenesis. Development.

[35] Furuta C., Miyamoto T., Takagi T., Noguchi Y., Kaneko J., Itoh S., Watanabe T., Itoh F. (2015). Transforming growth factor-β signaling enhancement by long-term exposure to hypoxia in a tumor microenvironment composed of Lewis lung carcinoma cells. Cancer Sci..

[36] Nakanishi Y., Kodama J., Yoshinouchi M., Tokumo K., Kamimura S., Okuda H., Kudo T. (1997). The expression of vascular endothelial growth factor and transforming growth factor-β associates with angiogenesis in epithelial ovarian cancer. Int. J. Gynecol. Pathol..

[37] Xiong B., Gong L.L., Zhang F., Hu M.B., Yuan H.Y. (2002). TGF β_1_ expression and angiogenesis in colorectal cancer tissue. World J. Gastroenterol..

[38] Seebauer C.T., Brunner S., Glockzin G., Piso P., Ruemmele P., Schlitt H.J., Geissler E.K., Fichtner-Feigl S., Kesselring R. (2016). Peritoneal carcinomatosis of colorectal cancer is characterized by structural and functional reorganization of the tumor microenvironment inducing senescence and proliferation arrest in cancer cells. OncoImmunology.

[39] Alix-Panabières C., Pantel K. (2014). Challenges in circulating tumour cell research. Nat. Rev. Cancer.

[40] Yu M., Stott S., Toner M., Maheswaran S., Haber D.A. (2011). Circulating tumor cells: approaches to isolation and characterization. J. Cell Biol..

[41] Gay L.J., Felding-Habermann B. (2011). Platelets and cancer cells: Contribution of platelets to tumour metastasis. Nat. Rev. Cancer.

[42] Yan M., Jurasz P. (2016). The role of platelets in the tumor microenvironment: From solid tumors to leukemia. Biochim. Biophys. Acta.

[43] Chua H.L., Bhat-Nakshatri P., Clare S.E., Morimiya A., Badve S., Nakshatri H. (2007). NF-κB represses E-cadherin expression and enhances epithelial to mesenchymal transition of mammary epithelial cells: potential involvement in metastasis. Cancer Res..

[44] Vestweber D. (2015). How leukocytes cross the vascular endothelium. Nat. Rev. Immunol..

[45] Morath I., Hartmann T.N., Orian-Rousseau V. (2016). CD44: More than a mere stem cell marker. Int. J. Biochem. Cell Biol..

[46] Senbanjo L.T., Chellaiah M.A. (2017). CD44: a multifunctional cell surface adhesion receptor is a regulator of progression and metastasis of cancer cells. Front. Cell Dev. Biol..

[47] Mohamadzadeh M., DeGrendele H., Arizpe H., Estess P., Siegelman M. (1998). Proinflammatory stimuli regulate endothelial hyaluronan expression and CD44/HA-dependent primary adhesion. J. Clin. Invest..

[48] Kawakami N., Nishizawa F., Sakane N., Iwao M., Tsujikawa K., Ikawa M., Okabe M., Yamamoto H. (1999). Roles of integrins and CD44 on the adhesion and migration of fetal liver cells to the fetal thymus. J. Immunol..

[49] Nandi A., Estess P., Siegelman M.H. (2000). Hyaluronan anchoring and regulation on the surface of vascular endothelial cells is mediated through the functionally active form of CD44. J. Biol. Chem..

[50] May T., Mueller P.P., Weich H., Froese N., Deutsch U., Wirth D., Kröger A., Hauser H. (2005). Establishment of murine cell lines by constitutive and conditional immortalization. J. Biotech..

[51] Levéen P., Larsson J., Ehinger M., Cilio C.M., Sundler M., Sjöstrand L.J., Holmdahl U., Karlsson S. (2002). Induced disruption of the transforming growth factor beta type II receptor gene in mice causes a lethal inflammatory disorder that is transplantable. Blood.

[52] Nakano N., Tsuchiya Y., Kako K., Umezaki K., Sano K., Ikeno S., Otsuka E., Shigeta M., Nakagawa A., Sakata N. (2017). TMED10 protein interferes with Transforming growth factor (TGF)-β signaling by disrupting TGF-β receptor complex formation. J. Biol. Chem..

