# Supplementary material for: Reduced lung metastasis in endothelial cell-specific transforming growth factor β type II receptor-deficient mice with decreased CD44 expression
Source: iScience. 2024 Nov 28;27(12):111502. doi: 10.1016/j.isci.2024.111502 (PMC11699617; doi:10.1016/j.isci.2024.111502)
Supplement: Document S1. Figures S1–S7 and Tables S1–S3 [file mmc1.pdf]

iScience, Volume *27*

Supplemental information

Reduced lung metastasis in endothelial cell-specific transforming growth factor β type II receptor-deficient mice with decreased CD44 expression

Kako Hanada, Yuki Saito, Takahiro Takagi, Mitsuki Go, Yota Nakano, Toshihiko Inagawa, Hideyo Hirai, Marcus Fruttiger, Susumu Itoh, and Fumiko Itoh

Figure S1.

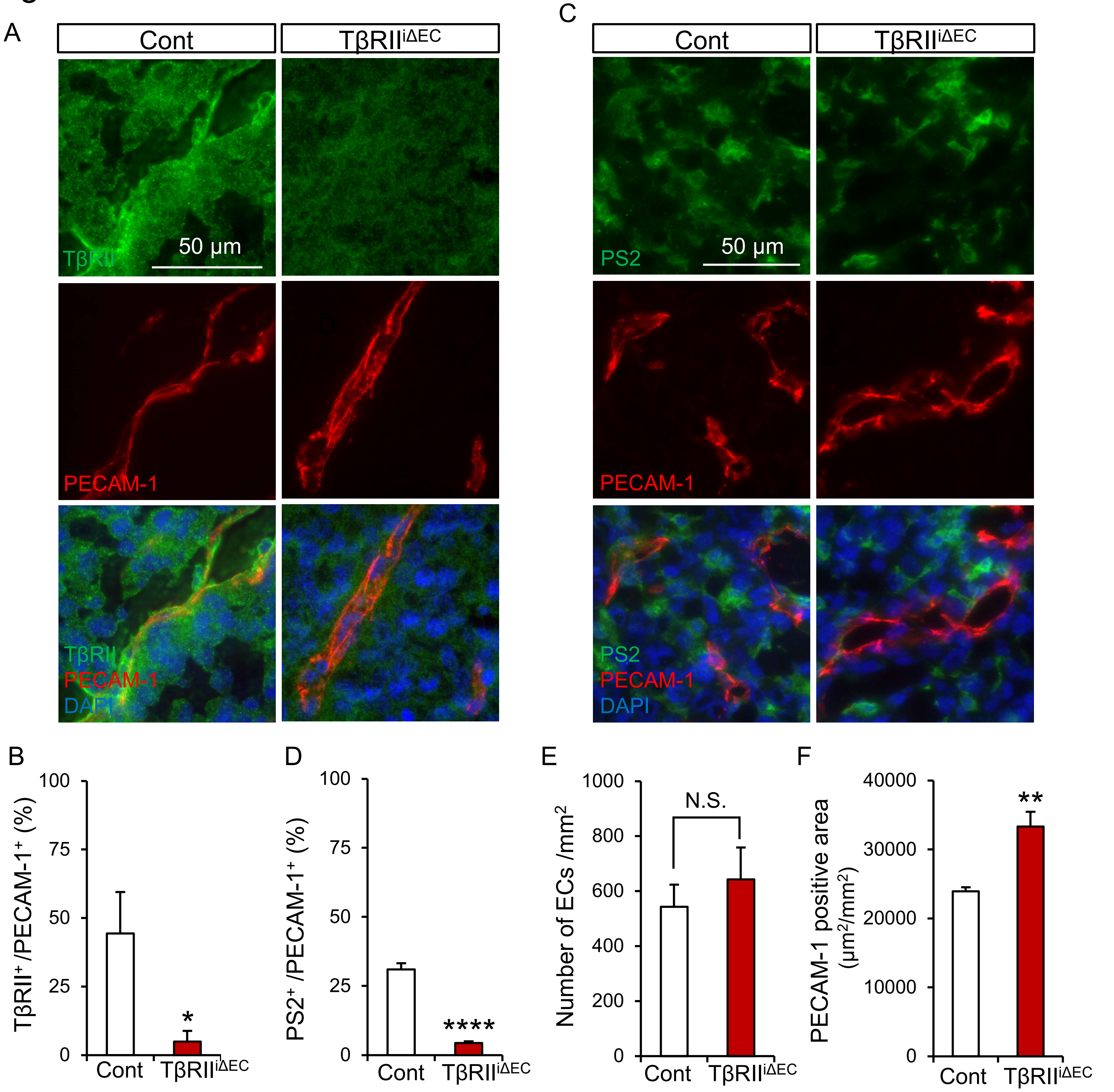

Supplementary Figure 1. Effects of TβRII depletion following tamoxifen administration in endothelial cells.

(**A**) immunofluorescence staining of tumor sections harvested at day 12 post-tamoxifen administration, using anti-TβRII and anti-PECAM-1 antibodies. Representative images from three independent mice are shown. (**B**) Quantitative analysis of the TβRII-positive area normalized to the PECAM-1-positive area. Data are presented as means ± SDs. Statistical comparisons were performed using an unpaired two-tailed t-test. n = 3 mice/group, with 2 images (1 mm² each) analyzed per mouse. *P < 0.05. (**C**) immunofluorescence staining for phosphorylated Smad2 (PS2) and PECAM-1 in tumor sections at day 12. (**D**) Quantitative analysis of PS2-positive areas, normalized to PECAM-1-positive areas, based on images from three independent mice, including the representative shown in (C). (unpaired two-tailed t-test, n=3 each, ****P< 0.0001) (**E and F**) Quantitative analysis of images from 3 mice, including the representative (C). The number of blood vessels (E), and PECAM-1-positive area (F) per unit area were measured. Data are presented as means ± SDs. Statistical comparisons were performed using an unpaired two-tailed t-test. n = 3 mice/group, with 2 images (1 mm² each) analyzed per mouse. **P< 0.001 , N.S.: not significant).

Figure S2.

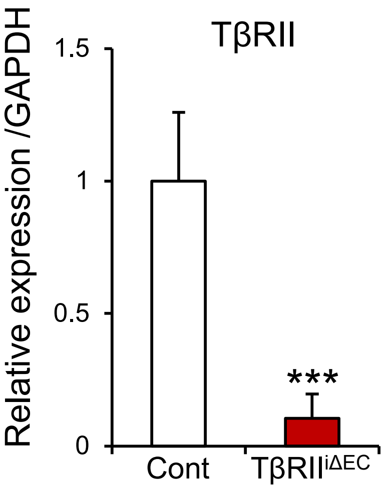

Supplementary Figure 2. Reduction of TβRII mRNA in lung endothelial cells following tamoxifen administration.

Lung vascular endothelial cells were isolated by fluorescence-activated cell sorting (FACS) using PE-conjugated anti-mouse CD309 (VEGFR2, Flk-1) antibody from control and tamoxifen-treated TβRII$^{i\Delta EC}$ mice 10 days post-tamoxifen administration. Quantitative PCR (qPCR) was performed to measure TβRII mRNA levels. The results show a significant reduction in TβRII mRNA expression in the tamoxifen-treated group (red bar) compared to the control group (white bar), indicating successful depletion of TβRII in endothelial cells following tamoxifen treatment. Data are presented as means ± SDs. Statistical comparisons were performed using an unpaired two-tailed t-test. n = 3 mice for the control group and n = 6 mice for the TβRII$^{i\Delta EC}$ group. ***$p<0.001$.

Figure S3.

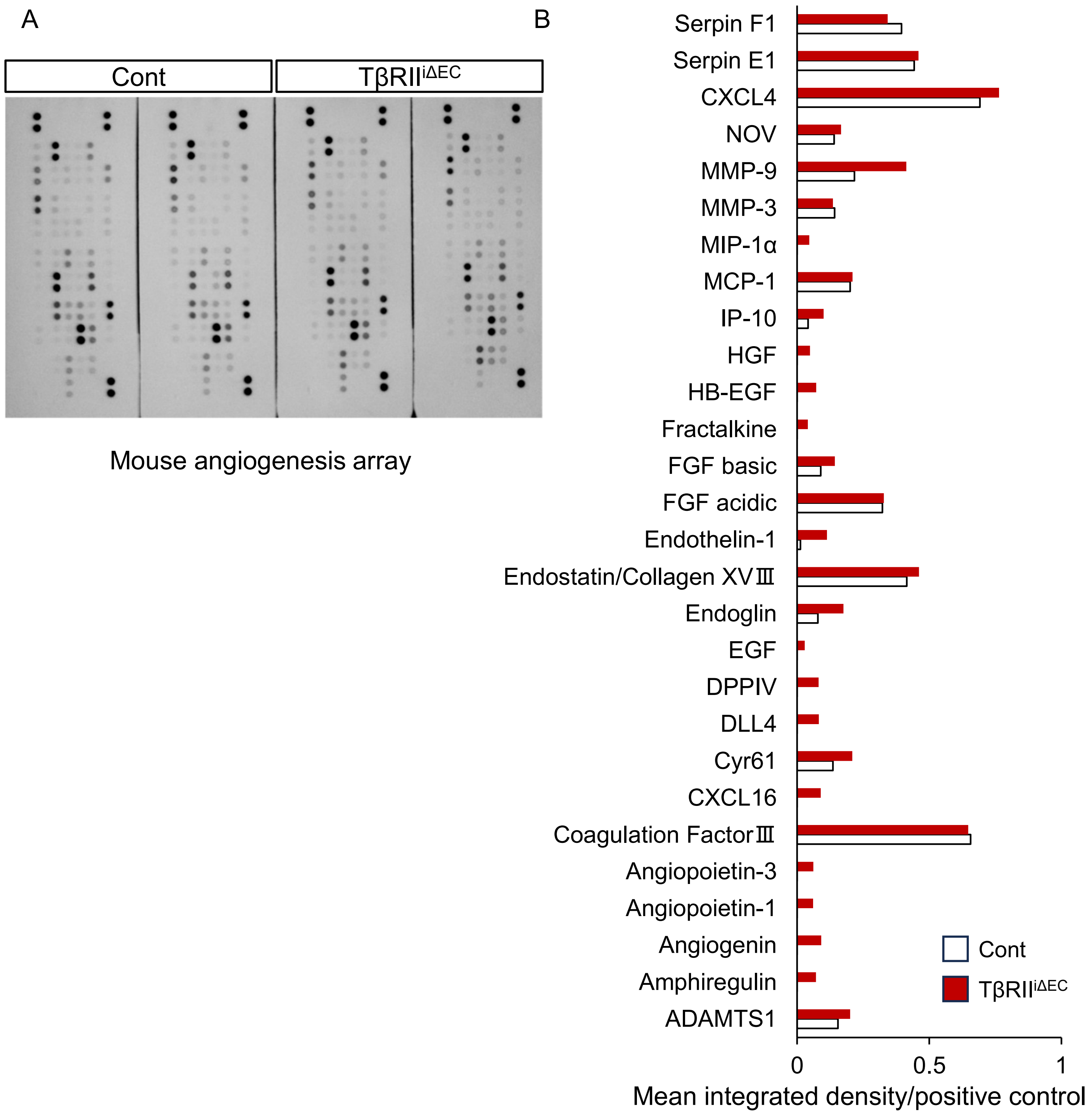

Supplementary Figure 3. Pro-angiogenic properties of tumor tissues.
(A) Two control and two TβRII$^{i\Delta EC}$ mice were implanted with LLC, and tumor tissues were harvested on day 12 for protein array analysis of angiogenesis-related cytokines. Angiogenic protein profiling was assessed using a mouse angiogenesis antibody array (R&D Systems) according to the manufacturer's instructions, and visualized with a LAS-4000 imaging system (Fujifilm). **(B)** Quantification of selected angiogenic factors was performed using ImageJ software, with the graphs representing the average values obtained from two mice per group. Some cytokines were excluded from the analysis due to low expression levels.

Figure S4.

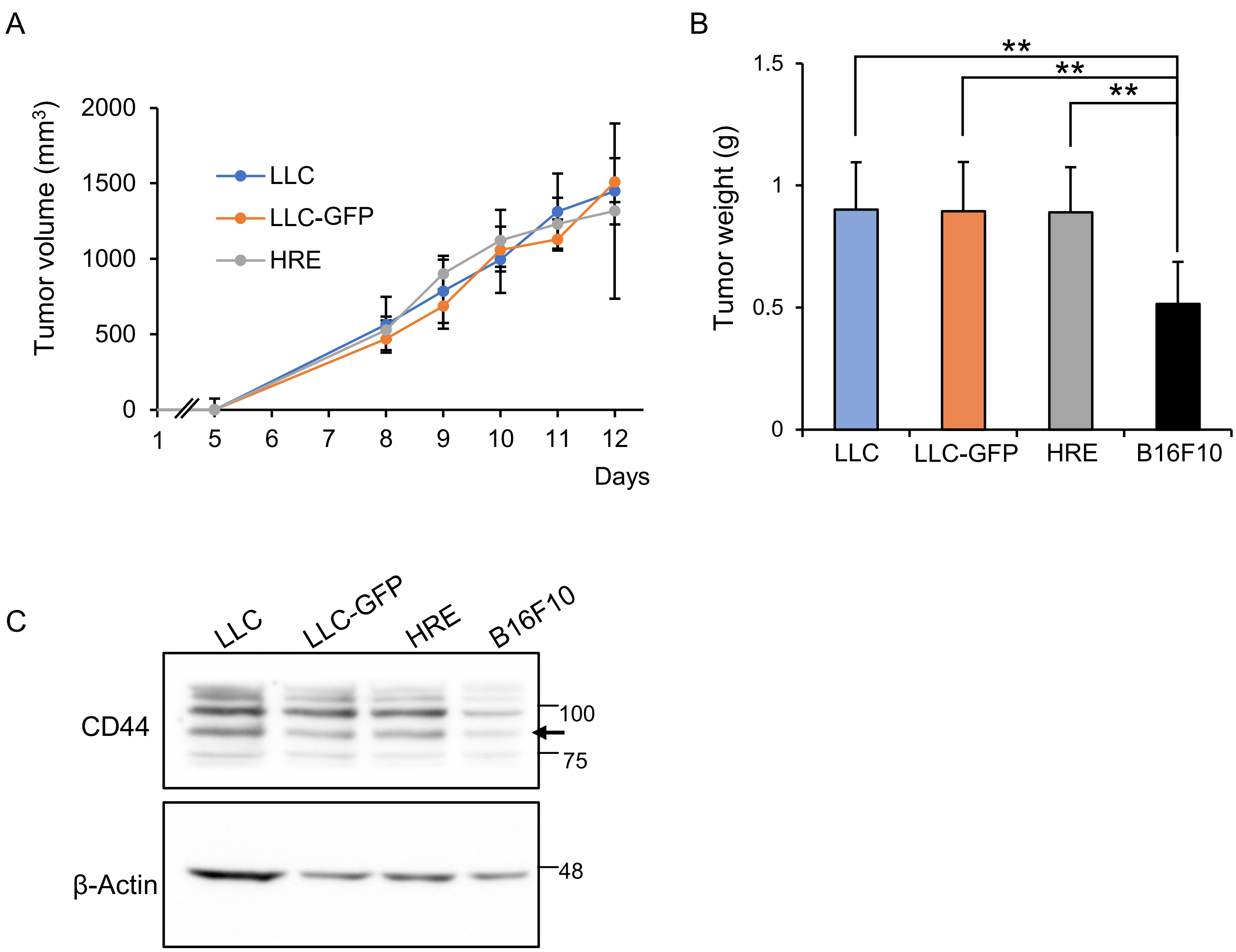

Supplementary Figure 4. Tumor growth and final tumor weight comparison among LLC, LLC-GFP, and LLC-p5HRE-EGFP.

(A) Tumor growth curves for LLC, LLC-GFP, and p5HRE-EGFP (HRE) cell lines in C57BL6 mice. Tumor volumes were measured over a period of 15 days. Data are presented as means ± SDs. Statistical comparisons were performed using ordinary 1-way ANOVA. n = 6 mice/group. No significant differences in tumor growth rates were observed among LLC, LLC-GFP, and HRE groups over the course of the experiment. **(B)** Quantification of tumor weight at the endpoint of the experiment. Tumors were excised and weighed, showing that the B16F10 tumors had a significantly lower weight compared to the other groups, while no significant differences were found among the LLC, LLC-GFP, and HRE groups. Data are presented as means ± SDs. Statistical comparisons were performed using ordinary 1-way ANOVA. (n=6 mice/group,**$p<0.01$.) **(C)** CD44 expression in LLC, LLC-eGFP, and p5HRE-EGFP (HRE) cell lines was assessed via Western blotting.

Figure S5.

A

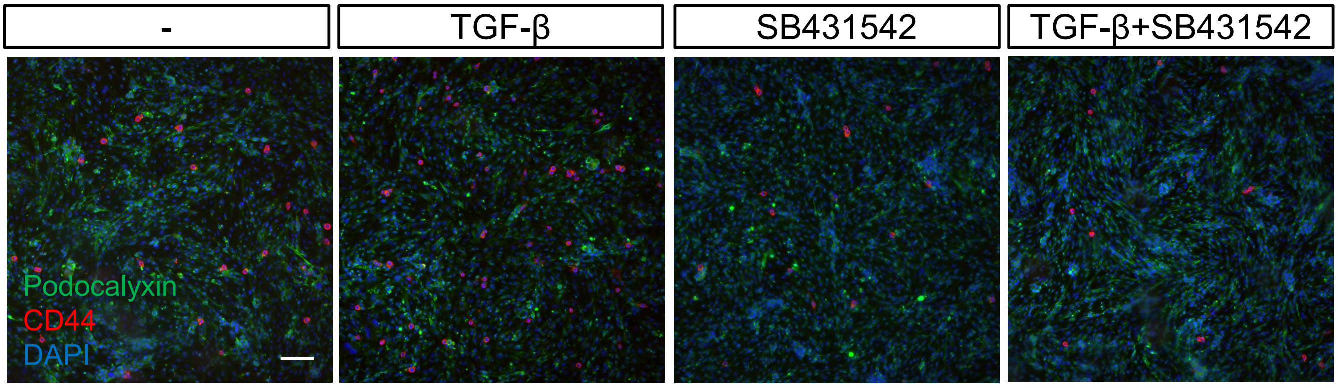

B

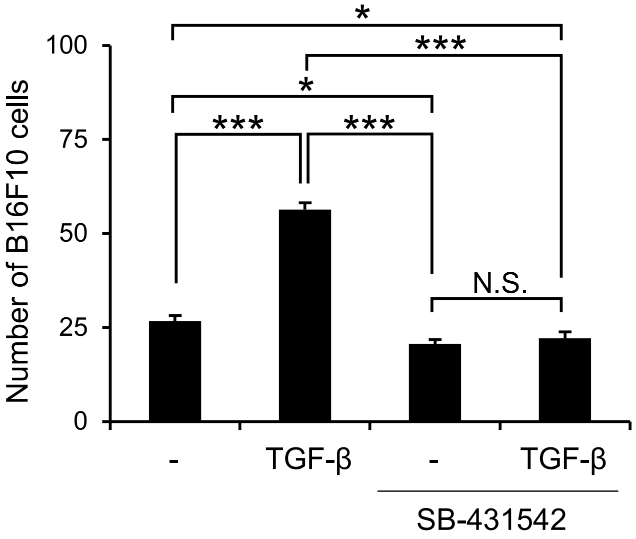

Supplementary Figure 6. Adhesion analysis of bEnd5 endothelial cells and B16F10 cells under TGF-β signaling.

(**A**) bEnd5 cells were cultured for 2 days with the addition of TGF-β (5 ng/mL), the ALK5 inhibitor SB-431542 (10 μmol/mL), or a combination of TGF-β and SB-431542. B16F10 cells were then introduced, and after 4 hours of coculture, the cells were immunostained with anti-podocalyxin and anti-CD44 antibodies. B16F10 cells were detectable due to their strong expression of CD44 (Figure S4). (**B**) A fluorescence microscope was used to quantify the number of B16F10 cells adherent to vascular endothelial cells per unit area. Data are presented as means ± SDs. (ordinary 1-way ANOVA, n=3 represents independent experiments, *P<0.05; ***P< 0.001, N.S.: not significant)

Figure S6.

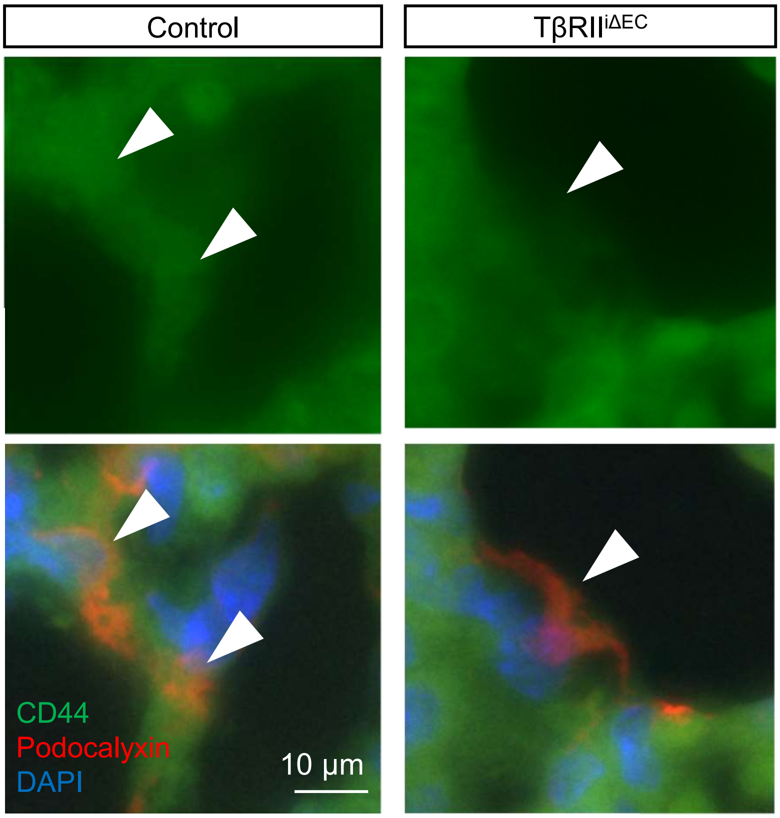

Supplementary Figure 6. Influence of endothelial TβRII deficiency on CD44 expression.

Immunofluorescence staining of paraffin-embedded lung sections one month after tamoxifen administration. CD44 expression was reduced in podocalyxin-positive regions (arrow head), which correspond to blood vessels stained in red, indicating vascular structures in $T\beta RII^{i\Delta EC}$ mice.

Figure S7.

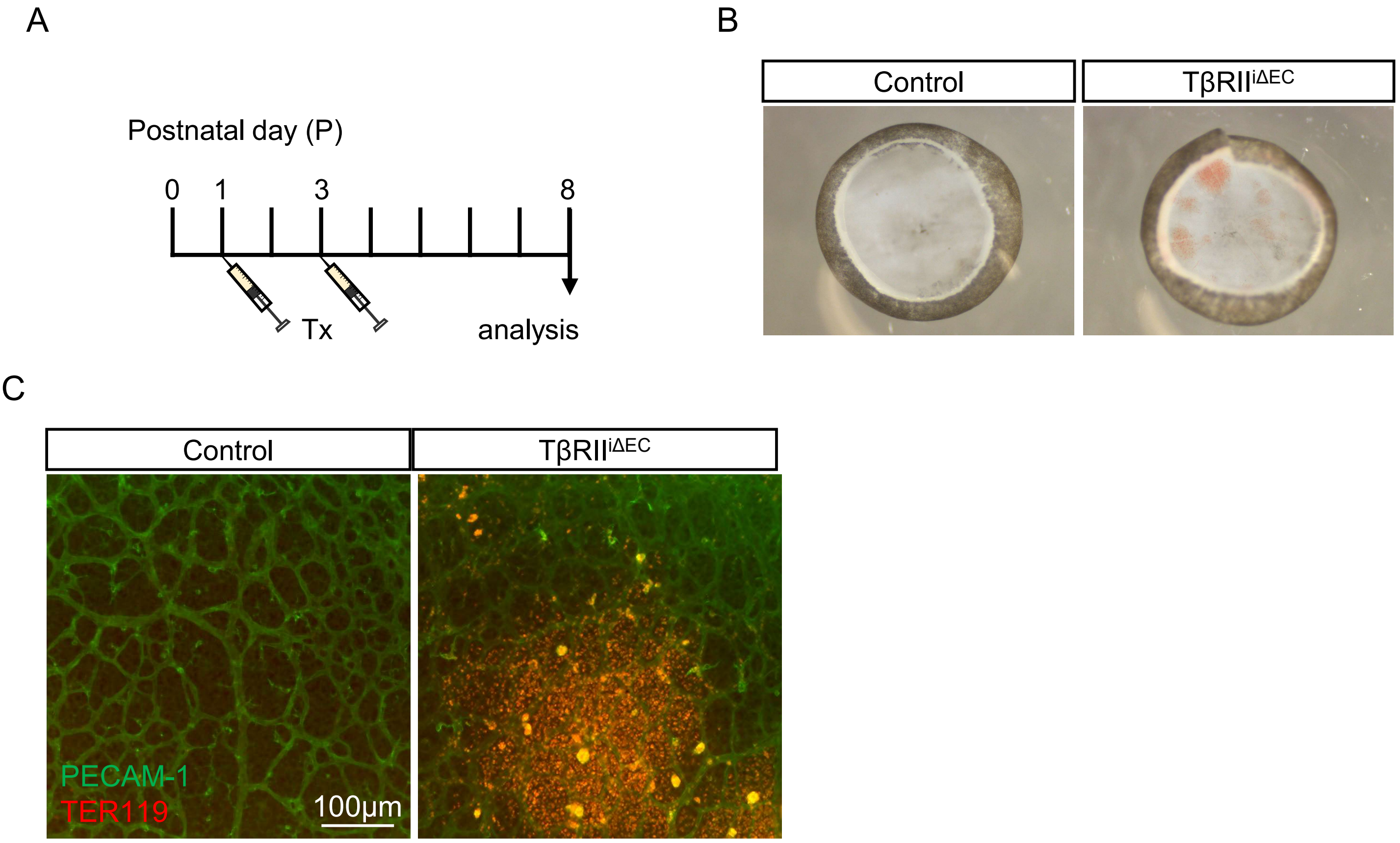

Supplementary Figure 7. Impact of selective inhibition of TβRII signaling on neonate angiogenesis.

(**A**) Experimental protocol for tamoxifen (Tx) injection. (**B**) Excised retina of control and TβRII$^{i\Delta EC}$ neonate (day 8). Hemorrhaging was observed in the TβRII$^{i\Delta EC}$ neonatal retina. (**C**) Immunofluorescence analysis of retinas using anti-PECAM-1 and anti-TER119 antibodies on postnatal day 8 (P8). Retinas were incubated in 4% paraformaldehyde (PFA) in phosphate-buffered saline (PBS) for 15–20 minutes at room temperature, then dissected and fixed with methanol at -25°C overnight. After washing with PBS containing 0.1% Tween 20, samples were incubated in Blocking Reagent (DAKO, Glostrup, Denmark) for 1 hour at room temperature. Primary antibodies diluted in Blocking Reagent were added and incubated overnight at 4°C. The samples were washed three times with 0.1% Tween 20/PBS, followed by incubation with Alexa Fluor-conjugated secondary antibodies (listed in Supplemental Table 1).

Supplemental Table 1. Antibody information.

Antibodies	Dilution	source	Identifier
rat monoclonal anti-PECAM-1 antibody	IF 1:200	BD Biosciences	Cat#550274
goat anti VEGFR3 antibody	IF 1:100	R&D systems	35917
Anti-Actin, alpha-Smooth Muscle-Cy3 ™ antibody	IF 1:200	Millipore Sigma	Cat#C6198
α-Smooth Muscle Actin Rabbit mAb	IF 1:400	Cell Signaling Technology	Cat#D4K9N
APC rat anti-mouse TER119/Erythroid Cells Antibody	IF 1:200	BD Pharmingen™	Cat#561033
Rabbit polyclonal pSmad2 Ab	IF 1:200	homemade	
Anti-TGF beta Receptor II antibody [EPR14673]	IF 1:200	abcam	ab184948
Anti-Ki67 antibody [SP6]	IF 1:200	abcam	ab16667
Cleaved Caspase-3 (Asp175) Antibody	IF 1:200	Cell Signaling Technology	Cat#9661
Monoclonal Anti-β-Actin, Clone AC-74	WB 1:5000	Sigma-Aldrich	Cat#A2228
Purified anti-mouse/human CD44 Antibody	WB 1:1000	BioLegend	Cat#103001
Mouse Podocalyxin Biotinylated Antibody	IF 1:200	R&D Systems	BAF1556
Alexa488-conjugated donkey anti-rabbit IgG	IF 1:200	Thermo Fisher Scientific	A21206
Alexa488-conjugated goat anti-rabbit IgG	IF 1:200	Thermo Fisher Scientific	A11008
Alexa594-conjugated goat anti-rat IgG	IF 1:200	Thermo Fisher Scientific	A21208
PE anti-mouse CD309 (VEGFR2, Flk-1) Antibody	FC 1:1000	BioLegend	Cat#136403
ECL™ Anti-mouse IgG, Horseradish Peroxidase-linked Whole Antibody (from sheep)	WB 1:10000	GE Healthcare Life Sciences	NA931V
ECL™ Anti-rat IgG, Horseradish Peroxidase-linked Whole Antibody (from goat)	WB 1:10000	GE Healthcare Life Sciences	NA935V

Supplemental Table 2. Primer sequence.

gene	primer sequence
GAPDH	(forward) 5'-TGCCGTTGAATTTGCCGT-3'<br>(reverse) 5'-TGCAGTGGCAAAGTGGAGATT-3'
VEGF-A	(forward) 5'-CCCACGTCAGAGAGCAACAT-3'<br>(reverse) 5'-CCGGGATTTCTTGCGCTTTC-3'
angiopoietin 1	(forward) 5'-CAGGCAAACAGAGCAGCTTG-3'<br>(reverse) 5'-CATCGAACCACCAACCTCCT-3'
TβRII	(forward) 5’-TGCATCCATCCACCTAAGCTG-3'<br>(reverse) 5’-TCCACAGGACGATATGCAGC-3'

Supplemental Table 2. Statistical information.

F.TEST, T.TEST				
**Figure No.**		**F values**	**T values**	**n values**
**1D**		0.533927306	0.803130769	6
**2B**		0.240394021	0.181769479	2 images x3
**2C**		0.96921848	0.002792232	2 images x3
**2D**		0.361726938	0.00333756	2 images x3
**2H**		0.366136265	0.982455228	2 images x3
**3C**	VEGF-A	0.493375411	0.023495984	6
	angiopoietin 1	0.952831257	0.045797662	6
**4B**		0.00333925	0.045973885	6
**4E**		0.459760036	2.36288E-06	6
**Figure No.**	**one-way ANOVA**			
**6C**	**F values**	**degrees-of-freedom**	**p values**	**n values**
	132.1285141	3-8	3.7395E-07	2 images x3
	**F.TEST, T.TEST**			
		**F values**	**T values**	**n values**
	Control vs TGF-β	0.383495146	0.001075584	2 images x3
	Control vs SB-431542	0.682997118	0.0015551	2 images x3
	Control vs TGF-β+SB-431542	0.718918919	0.000241234	2 images x3
	TGF-β vs SB-431542	0.627747253	0.000197392	2 images x3
	TGF-β vs TGF-β+SB-431542	0.234982332	9.07533E-05	2 images x3
	SB431543 vs TGF-β+SB-431542	0.450847458	0.202361366	2 images x3
**Figure No.**	**one-way ANOVA**			
**6E**	**F values**	**degrees-of-freedom**	**p values**	
	8.423210195	3-16	0.00137889	5
	**F.TEST, T.TEST**			
		**T values**		**n values**
	Control vs TGF-β	0.049180876		5
	Control vs SB-431542	0.030050306		5
	Control vs TGF-β+SB-431542	0.012497498		5
	TGF-β vs SB-431542	0.012765227		5
	TGF-β vs TGF-β+SB-431542	0.006528244		5
	SB431543 vs TGF-β+SB-431542	0.570965528		5
**Figure No.**	**one-way ANOVA**			
**6G**	**F values**	**degrees-of-freedom**	**p values**	
	9.752687283	2-6	0.013018431	2 images x3
	**F.TEST, T.TEST**			
		**F values**	**T values**	**n values**
	none vs Cont. Ab	0.442725535	0.861164396	2 images x3
	none vs anti-CD44	0.375119617	0.004009845	2 images x3
	Cont. Ab vs anti-CD44	0.123180057	0.022501005	2 images x3
**Figure No.**	**one-way ANOVA**			
**7C**	**F values**	**degrees-of-freedom**	**p values**	
	127.0676396	2-6	1.22703E-05	2 images x3
	**F.TEST, T.TEST**			
		**F values**	**T values**	**n values**
	Cont vs shCD44#1	0.918634403	0.000269969	2 images x3
	Cont vs shCD44#2	0.727106969	0.000100623	2 images x3
	shCD44#1 vs shCD44#2	0.653436504	0.282631457	2 images x3

Supplemental Table 3. Statistical information.

F.TEST, T.TEST				
**Figure No.**		**F values**	**T values**	**n values**
**S1B**		0.123940342	0.023143969	2 images x3
**S1D**		0.133189316	9.3912E-05	2 images x3
**S1E**		0.639680502	0.378104782	2 images x3
**S1F**		0.144662394	0.003889592	2 images x3
**S2**		0.059731437	0.000919158	6
**Figure No.**	**one-way ANOVA**			
**S4B**	**F values**	**degrees-of-freedom**	**p values**	**n values**
	5.136003705	3-20	0.0085226	6
	**F.TEST, T.TEST**			
		**F values**	**T values**	**n values**
	LLC vs LLC-GFP	0.882088114	0.954141378	6
	LLC vs HRE	0.964359989	0.918422398	6
	LLC vs B16F10	0.844443351	0.005223212	6
	LLC-GFP vs HRE	0.854176196	0.968212995	6
	HRE vs B16F10	0.884612418	0.00783834	6
	B16F10 vs LLC-GFP	0.742562831	0.00949345	6
**Figure No.**	**one-way ANOVA**			
**S5B**	**F values**	**degrees-of-freedom**	**p values**	**n values**
	230.9106383	3-20	0.00000004	2 images x3
	**F.TEST, T.TEST**			
		**F values**	**T values**	**n values**
	Control vs TGF-β	0.81038961	5.4266E-05	2 images x3
	Control vs SB-431542	0.795366795	0.01052912	2 images x3
	Control vs TGF-β+SB-431542	0.836461126	0.046499503	2 images x3
	TGF-β vs SB-431542	0.620481928	1.92927E-05	2 images x3
	TGF-β vs TGF-β+SB-431542	0.97309417	4.11006E-05	2 images x3
	SB-431542 vs TGF-β+SB-431542	0.64375	0.371234344	2 images x3
